# Prediction of GluN2B-CT_1290-1310_/DAPK1 Interaction by Protein–Peptide Docking and Molecular Dynamics Simulation

**DOI:** 10.3390/molecules23113018

**Published:** 2018-11-19

**Authors:** Gao Tu, Tingting Fu, Fengyuan Yang, Lixia Yao, Weiwei Xue, Feng Zhu

**Affiliations:** 1 Innovative Drug Research and Bioinformatics Group, School of Pharmaceutical Sciences and Collaborative Innovation Center for Brain Science, Chongqing University, Chongqing 401331, China; tugao@cqu.edu.cn (G.T.); 20162902031@cqu.edu.cn (T.F.); kristen@cqu.edu.cn (F.Y.); 2 Innovative Drug Research and Bioinformatics Group, College of Pharmaceutical Sciences, Zhejiang University, Hangzhou 310058, China; 3 Department of Health Sciences Research, Mayo Clinic, Rochester, MN 55905, USA; ballad2006@163.com

**Keywords:** DAPK1-GluN2B peptide, protein–peptide docking, MD simulation, binding free energy, hotspot

## Abstract

The interaction of death-associated protein kinase **1** (DAPK1) with the 2B subunit (GluN2B) C-terminus of N-methyl-D-aspartate receptor (NMDAR) plays a critical role in the pathophysiology of depression and is considered a potential target for the structure-based discovery of new antidepressants. However, the 3D structures of C-terminus residues 1290–1310 of GluN2B (GluN2B-CT_1290-1310_) remain elusive and the interaction between GluN2B-CT_1290-1310_ and DAPK1 is unknown. In this study, the mechanism of interaction between DAPK1 and GluN2B-CT_1290-1310_ was predicted by computational simulation methods including protein–peptide docking and molecular dynamics (MD) simulation. Based on the equilibrated MD trajectory, the total binding free energy between GluN2B-CT_1290-1310_ and DAPK1 was computed by the mechanics generalized born surface area (MM/GBSA) approach. The simulation results showed that hydrophobic, van der Waals, and electrostatic interactions are responsible for the binding of GluN2B-CT_1290–1310_/DAPK1. Moreover, through per-residue free energy decomposition and in silico alanine scanning analysis, hotspot residues between GluN2B-CT_1290-1310_ and DAPK1 interface were identified. In conclusion, this work predicted the binding mode and quantitatively characterized the protein–peptide interface, which will aid in the discovery of novel drugs targeting the GluN2B-CT_1290-1310_ and DAPK1 interface.

## 1. Introduction

It has been estimated that depression will be the second largest global health burden among all disorders by 2030 [1,2,3]. Current pharmacotherapies are mainly monoaminergic-acting antidepressants including the selective serotonin reuptake inhibitors (SSRIs), norepinephrine reuptake inhibitors (sNRIs), serotonin/norepinephrine reuptake inhibitors (SNRIs), and the tricyclic antidepressants amitriptyline and monoamine oxidase inhibitors [4,5,6,7]. However, these medications are not ideal considering the necessity of prolonged administration for clinical improvement, which makes the discovery of rapidly acting antidepressants with novel mechanism an urgent task [8,9,10,11,12]. The interaction between death-associated protein kinase **1** (DAPK1) and the C-terminus of the 2B subunit (GluN2B) of the N-methyl-D-aspartate receptor (NMDAR) is reported to potentiate the activity of NR2B-containing NMDAR (NR2BR) [13,14,15]. Through triggering the DAPK1-mediated phosphorylation of NR2B subunit at Ser-1303, it induces injurious Ca^2+^ influx through NMDA receptor channels and leads to irreversible neuronal death [13,14,15]. The interaction between DAPK1 and GluN2B in the medial prefrontal cortex was recently found to contribute to the development of depressive-like behavior [16,17], and several studies have confirmed that DAPK1 is a promising target for the treatment of strokes, especially to prevent neuronal apoptosis in neuronal cell death [18,19,20].

As a serine/threonine kinase, DAPK1 has been found to induce programmed cell death by mediating gamma-interferon [21,22], and it is hypothesized to play a role in perinatal brain injury [23]. In the meantime, GluN2B, which primarily resides in extrasynaptic sites, is reported to be involved in cell death [24]. To regulate GluN2B’s conductance at extrasynaptic sites, activated DAPK1 directly binds to the C-terminal residues 1290–1310 of GluN2B (GluN2B-CT_1290-1310_) and phosphorylates the receptor at Ser1303 [25,26]. Preclinical studies have shown that the inhibition of DAPK1 and its interaction with GluN2B subunit exerts rapid antidepressant-like effects, suggesting the interaction between DAPK1 and GluN2B as a potential target for structure-based discovery of novel antidepressants [27,28,29,30]. So far, the 3D crystal structure of DAPK1 (residues 1–312) has already been determined at a resolution of 2 Å in its active form [17]. Regarding GluN2B, although several crystal structures have been released [31,32,33,34], the three-dimensional structure of GluN2B-CT_1290-1310_ remains elusive. Thus, the molecular mechanism of the GluN2B-CT_1290-1310_/DAPK1 interaction at the atomic level is still unknown and understanding of the GluN2B-CT_1290-1310_/DAPK1 interaction is urgently needed.

**Figure 1:**
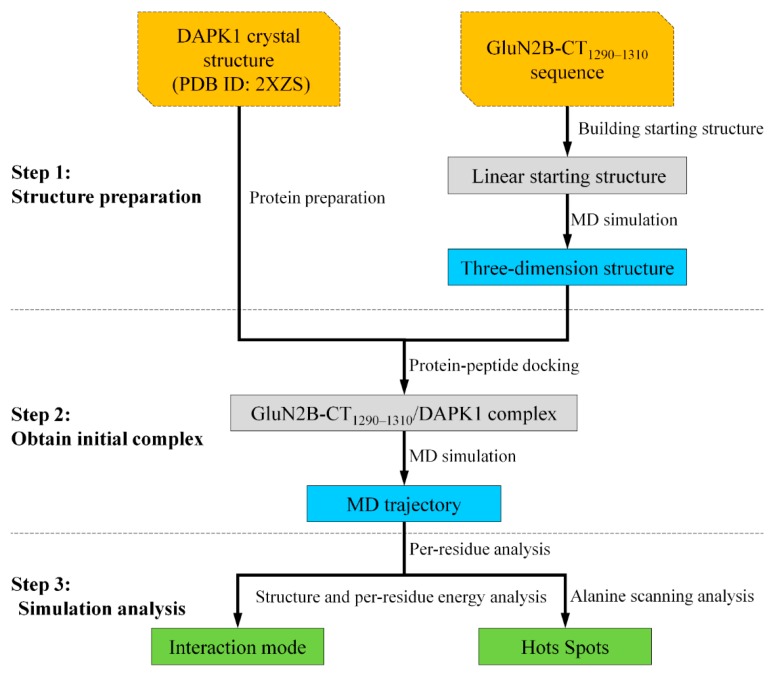
Flowchart of predicting GluN2B-CT_1290-1310_/DAPK1 interaction in this work. DAPK1: death-associated protein kinase **1**; MD: molecular dynamics.

In this study, a combined strategy integrating both protein–peptide docking and molecular dynamics (MD) simulation was employed to reveal the interaction mechanism between DAPK1 and GluN2B-CT_1290-1310_ (Figure 1). First, a three-dimensional structure of GluN2B-CT_1290-1310_ was constructed and optimized using MD simulation. Then, the initial pose of GluN2B-CT_1290-1310_ bound to DAPK1 was predicted by protein–peptide docking and assessed by MD simulation and a binding free energy calculation of the explicit solvent and molecular mechanics generalized born surface area (MM/GBSA). Finally, the binding free energy of each residue contributing to the studied interaction was obtained by per-residue energy decomposition analysis. Moreover, the hot spot residues (located within the interacting interface) contributing significantly to this interaction were identified by in-silico alanine scanning. As a result, this study reveals the mechanism underlying the GluN2B-CT_1290-1310_/DAPK1 interaction and provides insights into the structure-based discovery of drugs with this novel binding mechanism.

## 2. Results and Discussion

### 2.1. The Modeled Structure of the GluN2B C-Terminal Peptide

**Figure 2:**
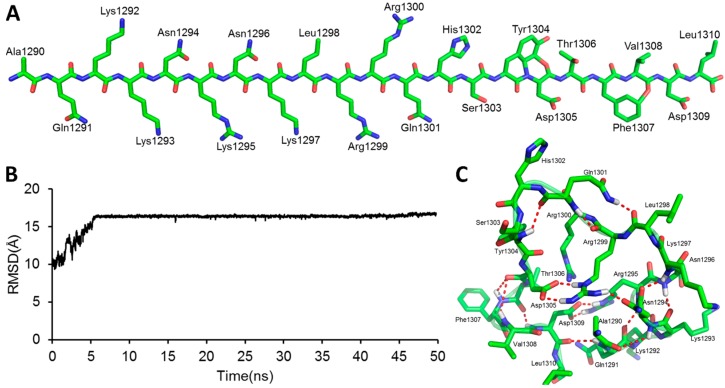
(**A**) Results of the starting linear peptide structure prediction of GluN2B-CT_1290-1310_. (**B**) The all-atoms root-mean-square-deviation (RMSD) was calculated with respect to the initial structure during the 50 ns trajectory A MD simulation. (**C**) The representative structure was generated by cluster analysis of 100 snapshots taken from the period of 40–50 ns of the trajectory A MD simulation. The twenty-one C-terminal residues of the GluN2B-CT_1290–1310_ are represented by green sticks. The red dashed lines represent the hydrogen bonds.

**Figure 3:**
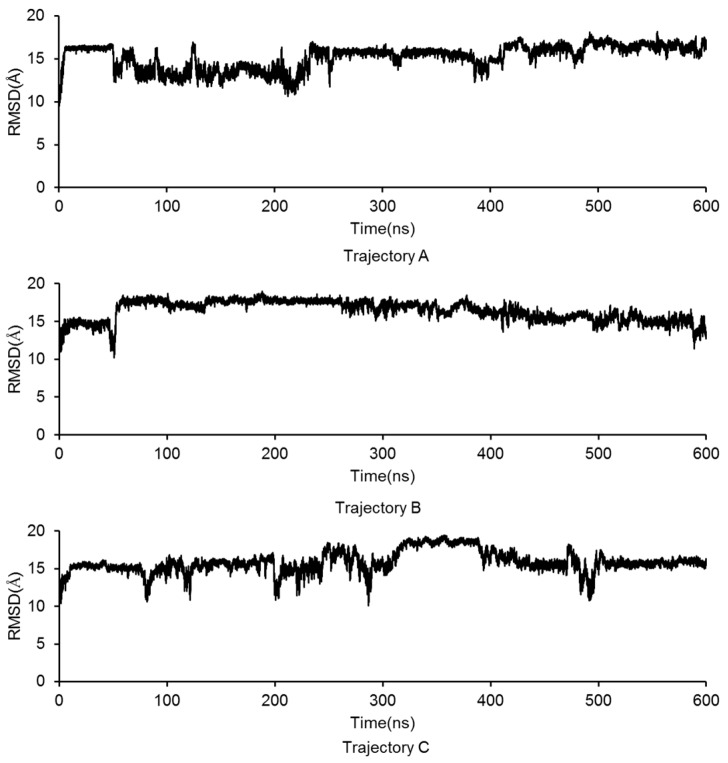
The GluN2B-CT_1290-1310_ all-atoms RMSD values along the 600 ns MD simulations for the repeated trajectories **A**, **B**, and **C**.

**Figure 4:**
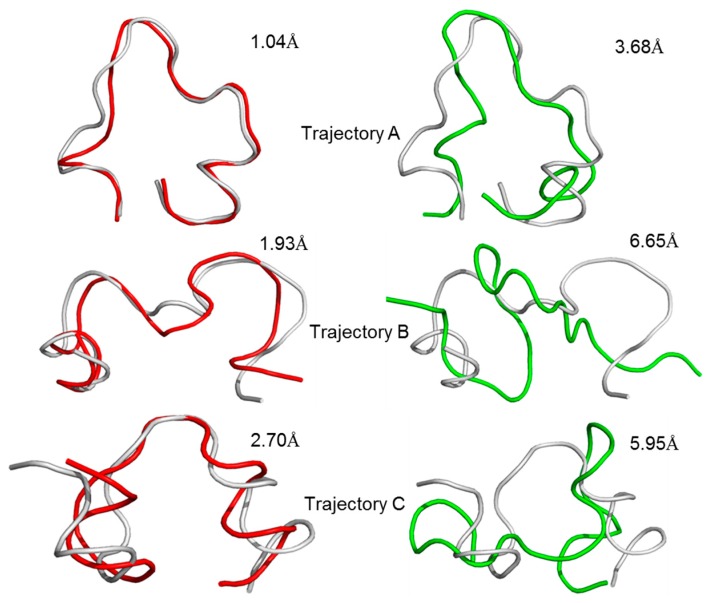
Representative structures and the calculated RMSD values of the representative structures between the first 10 ns (gray) and 50 ns (red), the first 10 ns and the last 100 ns (green) for the repeated trajectories **A**, **B** and **C**.

A linear GluN2B C-terminal peptide chain containing 21 amino acids (GluN2B-CT_1290-1310_) was first built using LEaP [35], and the two ends of the chain were terminated by methoxyl (N-terminus) and acetyl (C-terminus) (Figure 2A). To predict the three-dimensional structure of GluN2B-CT_1290-1310_, a 600-ns all-atoms MD simulation was carried out within the implicit solvent environment. The root-mean-square-deviation (RMSD) values of all-atoms along the first 50 ns of the trajectory A MD simulation are shown in Figure 2B. A representative snapshot of the 50 ns simulation of GluN2B-CT_1290-1310_ trajectory A was extracted (Figure 2C). It was observed that the peptide underwent a large degree of fluctuation (the RMSD reached ~16 Å) within the first 50 ns of three independent simulations, indicating that the fully extended conformation transformed from a stretched conformation to a collapsed random coil. Figure 2C illustrates that the internal hydrogen bond network was formed based on a number of residues (Gln1291, Arg1295, Lys1293, Asn1294, Lys1297, Arg1299, Gln1301, Tyr1304, Asp1305, Phe1307, Val1308, and Asp1309). In order to explore the native conformation of GluN2B-CT_1290-1310_, an additional 550 ns simulation was performed for trajectory A, and the RMSD values of all-atoms relative to the starting structure along the simulation time are shown in Figure 3. In addition, starting from the stretched structure, another two parallel (trajectories B and C) 600-ns MD simulations of the studied peptide were performed (Figure 3). Figure 4 demonstrates the extracted representative structure of each trajectory for the first 10 ns (gray) and 50 ns (red) and the last 100 ns (green). In order to monitor the structure transition from collapsed to near native conformation ensemble, the all-atoms RMSD of the representative structures between the first 10 ns and 50 ns, and the first 10 ns and the last 100 ns were calculated and labeled. The relatively larger RMSD between the first 10 ns and the last 100 ns illustrates that a relative long MD simulation (a total of 1.8 μs) is capable of capturing the near native conformation ensemble. Moreover, RMSD graphs were relatively stable over the last 100 ns of the conducted simulation, and the interaction detail of the representative snapshot of the last 100 ns MD simulation for each trajectory (Figures S1–S3) is provided in the “*The Modeled Structure of GluN2B C-Terminal Peptide*” section of the Supplementary Information. All three representative structures extracted from the final 100 ns of each equilibrated trajectory were selected as initial conformations for further protein–peptide docking and MD refinement.

### 2.2. Docking of GluN2B-CT_1290-1310_ into the DAPK1 Active Site

The initial structures of the representative conformations of GluN2B-CT_1290-1310_ into DAPK were obtained by different docking software packages, including *GRAMM-X docking* [36], *ZDOCK* [37] and *SwarmDock* [38]. For each software package, the top 10 docked poses were retained, and the results showed that GluN2B-CT_1290-1310_ was docked into different binding sites of DAPK1 (all docking poses were provided in Figures S4–S13). The structure of DAPK1 from the Protein Data Bank(PDB) database( PDB code: 2XZS) [17] contains Ser/Thr phosphorylation sites. Based on the experimental results [39,40,41], protein–peptide docking complexes were selected for further study based on the poses of peptides docked into or close to the phosphorylation ATP binding pocket of DAPK1 (Table 1). In addition, by considering both the resolution (less than 2 Å) and the species (human), another two DAPK1 crystal structures (PDB code: 5AV4 and 1JKS) were also selected for docking study by GRAMM-X docking. However, the results showed that there was no proper binding pose of the peptides in 5AV4 and 1JKS (Figures S14–S19). Thus, three DAPK1 crystal structures (PDB codes: 2XZS, 5AV4 and 1JKS) were aligned and are provided in Figure S20. As illustrated, there were significant conformation variations between the glycine-rich loops and the basic loops of 5AV4 and 1JKS with 2XZS, especially regarding the side chains of the activation loop residue Lys175 and the glycine-rich loop residue Gln23 which occupy the gates of active sites, impeding the docking of peptides onto the ATP binding pocket of DAPK1.

### 2.3. Molecular Dynamics Simulation of the GluN2B-CT_1290-1310_/DAPK1 Complex

**Figure 5:**
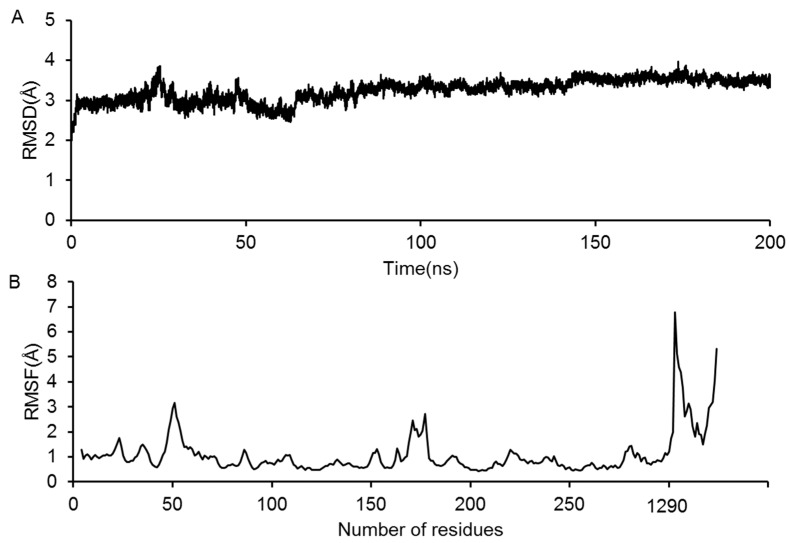
(**A**) The monitored root-mean-square-deviation (RMSD) of the all atoms with respect to the initial structure during the 200 ns simulation. (**B**) The root-mean-square-fluctuation (RMSF) value of backbone atoms of complex **1** during the 200 ns simulation.

Eight representative GluN2B-CT_1290-1310_/DAPK1 complexes predicted by the above docking were selected and subjected to further MD simulation. Taking the trajectory of predicted GluN2B-CT_1290-1310_/DAPK1 complex **1** as an example, the RMSDs of the all-atoms with respect to the initial structure during the 200 ns simulation were calculated and are shown in Figure 5A. The RMSD values were maintained at a level of 3 Å after 100 ns (Figure 5A), indicating that predicted GluN2B-CT_1290-1310_/DAPK1 complex **1** is nearly constant; the simulation trajectory converged within 200 ns. Additionally, to quantitatively monitor the fluctuations of each residue, the root-mean-square-fluctuation (RMSF) value of the predicted complex **1** was calculated over the 200 ns MD simulation (Figure 5B). The higher value of the peptide domain (residues 1290–1301) suggested that GluN2B-CT_1290-1310_ fluctuated significantly and underwent large conformational change to form a more stable interaction during the simulation (Figure 5B). The calculated RMSD and RMSF values for predicted complexes **2**–**8** (Figures S21 and S22) are shown in the “*Molecular Dynamics Simulation of GluN2B-CT_1290-1310_/DAPK1 Complexes*” section of the Supplementary Information. However, for predicted complexes **2** and **6**, the RMSD values jumped drastically within the 200 ns simulation (Figure S21A,E). An analysis of the representative structures showed that the peptide escapes from the DAPK1 phosphorylation of the ATP binding pocket. It was proposed that the incorrect initial conformation of predicted complexes **2** and **6** was not reasonable, since this kind of rare event would not occur in classic MD simulations. Therefore, predicted complexes **2** and **6** were not considered for further binding mode and free energy analyses.

### 2.4. Calculation of GluN2B-CT_1290-1310_/DAPK1 Binding Free Energy

To investigate the thermodynamic properties of protein–peptide interactions, a total of 500 snapshots extracted from the last 100 ns of the MD trajectory were used to calculate the MM/GBSA binding free energies. In predicted GluN2B-CT_1290-1310_/DAPK1 complex **1**, the major favorable contributor (−481.09 kcal/mol) to the protein–peptide was the electrostatic energy term (ΔE_ELE_), whereas the polar solvation energy (GB) to the solvation free energy term (ΔGB_TOT_) was unfavorable (509.81 kcal/mol) for the interaction. The van der Waals energy (ΔE_vdW_) for predicted GluN2B-CT_1290-1310_/DAPK1 complex **1** was −74.59 kcal/mol, which is a favorable contribution (Table 2). Moreover, the ΔE_ELE_ of predicted complexes **3**, **4**, **5**, **7,** and **8** showed that they are also major favorable contributors to the binding free energy (–635.30, −460.15, −427.51, −592.21, −606.89 kcal/mol, respectively, as shown in Tables S1–S5). In addition, the ΔE_vdW_ was shown to be an important energy contributor to the protein–peptide complex, as shown in Tables S1–S5 of the “*Calculation of GluN2B-CT1290-1310/DAPK1 Complexes Binding Free Energy*” section of Supplementary Information. The results of the calculated binding free energy for the six bound complexes suggested that both ΔE_ELE_ and ΔE_vdW_ determine the binding affinity of GluN2B-CT_1290-1310_ to the interface of DAPK1.

### 2.5. Analysis of Interactions between GluN2B-CT_1290-1310_ and DAPK1

#### 2.5.1. Insight from Free Energy Decomposition Analysis

**Figure 6:**
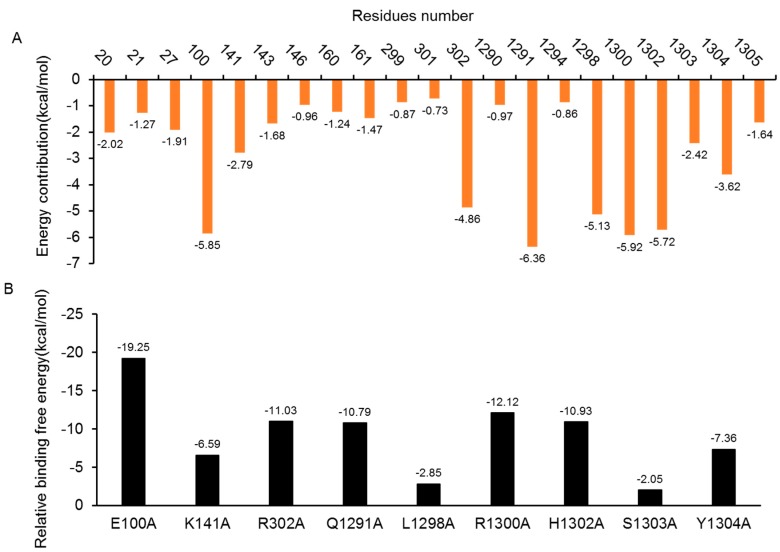
(**A**) Per-residue binding energy decomposition of predicted GluN2B-CT_1290–1310_/DAPK1 complex **1**. The energy contribution (the absolute value) larger than 0.60 kcal/mol to at least one of the studied residues for the binding of GluN2B-CT_1290–1310_/DAPK1 are displayed. The orange bar shows the residues with an absolute binding free energy value of more than 0.60 kcal/mol. (**B**) Alanine scanning analyses of predicted GluN2B-CT_1290–1310_/DAPK1 complex **1**.

To identity the interaction profile between the DAPK1 and GluN2B-CT_1290-1310_ interfaces, a per-residue binding free energy decomposition analysis was performed to determine the individual binding free energy. In predicted GluN2B-CT_1290-1310_/DAPK1 complex **1**, the contributions of 21 major residues (>0.60 kcal/mol) of the complex, which composed the primary portion (78%) of the total binding free energy, were compared and plotted in Figure 6A. The absolute energy contributions of residues Gly20, Glu100, Lys141, Arg302, Gln1291, Leu1298, Arg3100, His3102, Ser1303, and Tyr1304 were larger than 2 kcal/mol, especially for the residues Glu100, Gln1291 and Arg1300, whose corresponding energy contributions were −5.85, −6.36, and −5.92 kcal/mol, respectively. In addition, the free energy decomposition analysis of predicted complexes **3**, **4**, **5**, **7** and **8** is provided in the “*Free Energy Decomposition Analysis of GluN2B-CT_1290-1310_/DAPK1 Complexes*” section of the Supplementary Information (Figures S23A and S27A)

#### 2.5.2. Insights from the In-Silico Alanine Scanning Analysis

**Figure 7:**
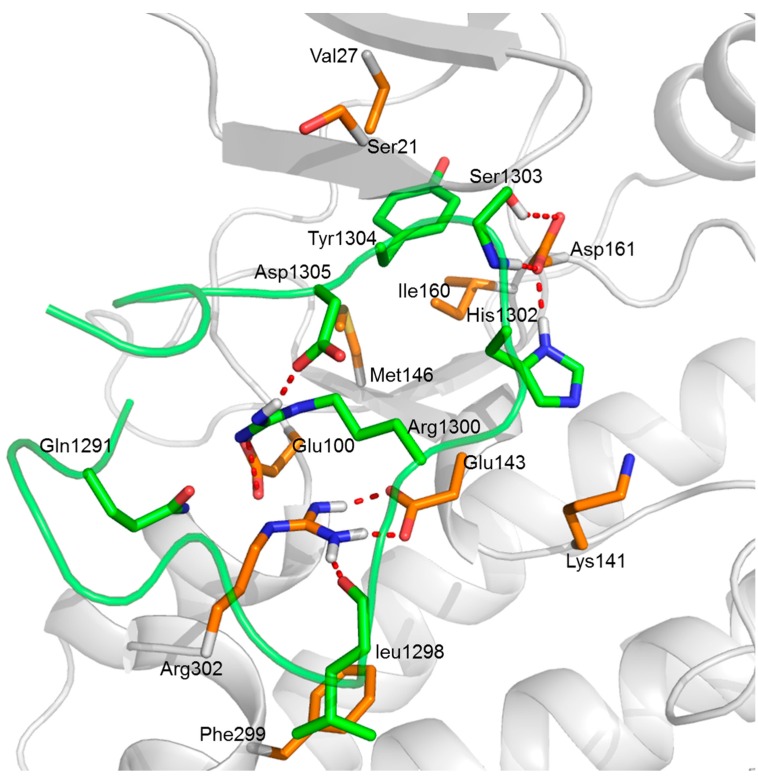
The predicted key interactions between the DAPK1 protein and the GluN2B-CT_1290–1310_ peptide in predicted complex **1**. The DAPK1 is shown as a grey cartoon while the GluN2B-CT_1290–1310_ is shown as a green cartoon. The C-terminal residues of the DAPK1 and the GluN2B-CT_1290–1310_ binding site residues are represented by orange and green sticks, respectively. The red dashed lines represent the hydrogen bonds.

The results of the in-silico alanine scanning for the selected nine residues in the predicted GluN2B-CT_1290-1310_/DAPK1 complex **1** interface are illustrated in Figure 6B. It is known that the residues that contribute >2 kcal/mol to the total binding free energy of the complex after mutating to an alanine are defined as hotspot residues [42,43,44,45,46,47,48]. As demonstrated in Figure 6B, nine residues (Glu100, Lys141, Arg302, Gln1291, Leu1298, Arg3100, His3102, Ser1303 and Tyr1304) were shown to contribute significantly (≥2 kcal/mol) to the binding between DAPK1 and GluN2B-CT_1290-1310_. Among these, Asp161 and His1302, were shown to be important for the binding of the interfaces, while Glu100 was shown to play a significant role for the DAPK1–peptide interfaces which was critical due to the size and conformation (Table 3 and Figure 7). The hotspot residue Glu100 demonstrated great impacts on the binding free energy difference (ΔΔG = −19.25 kcal/mol), and the interaction with Arg1300 formed hydrogen bonds to stabilize the peptide N-terminal. The salt bridge between Arg1300 and Asp1305 was shown to be critical for maintaining peptide structures. These hotspot residues contribute greatly to the stabilization of GluN2B-CT_1290-1310_/DAPK1 by hydrogen interactions, hydrophobic interactions, and, predominantly, through electrostatic interactions. The results of the in-silico alanine scanning analysis for predicted complexes **3**, **4**, **5**, **7** and 8 are provided in “*In Silico Alanine Scanning Analysis of GluN2B-CT_1290-1310_/DAPK1 Complexes*” in the Supplementary Information (Figures S23B–S27B)

#### 2.5.3. Insights from the Hydrogen Bond Interactions Network Analysis

The persistence of identified hydrogen bond with time during the MD simulation is demonstrated in Table 3. In predicted complex **1**, a strong electrostatic attraction with an average distance <3.50 Å between Arg302 and Glu143 was observed (occupying >45% during MD simulation). For this case, several hydrogen bonds are provided, as shown in Figure 7. Based on the analytical result, the peptide residues His1302 and Ser1303 were found to form hydrogen bonds with the residue Asp161 in the ATP binding pocket of DAPK1; the peptide residue Arg1300 was also found to engage in the hydrogen bond interactions with not only the peptide residue Asp1305 but also the DAPK1 residue Glu100. Furthermore, hydrophobic residues (Val27, Ile160 and Met146) within the hydrophobic pocket of DAPK1 were shown to interact with the peptide residue Tyr1304 via hydrophobic interactions (Figure 7). Therefore, electrostatic interactions and van der Waals interactions played the major roles in the interactions between GluN2B-CT_1290-1310_ and DAPK1. The hydrophobic pocket also contributed to the interactions. In addition, the results of the hydrogen bond interactions network analysis for predicted complexes **3**, **4**, **5**, **7** and **8** can be found in the “*Hydrogen Bond Interactions Network Analysis of GluN2B-CT_1290-1310_/DAPK1 Complexes*” section of the Supplementary Information (Figures S28–S32 and Tables S6–S10).

### 2.6. Identification of the Interface Profile of the GluN2B-CT_1290-1310_/DAPK1 Complex

The results of the analyses of interactions between GluN2B-CT_1290-1310_ and DAPK1 are shown in Table 4. After comparing the MD simulation results of predicted complexes **1**, **3**, **4** and **5**, it became clear that the common interface residues (His1302, Ser1303, and Tyr1304) in GluN2B-CT_1290-1310_ are in the vicinity of phosphorylation ATP binding pocket (Figure 7, and Figures S28–S30). In particular, in predicted complexes **1**, **4** and **5**, the hydrophobic interactions between GluN2B-CT_1290-1310_ Tyr1304 and the DAPK1 hydrophobic pocket (Ieu19, Val27and Met146) play crucial roles in protein–peptide recognition (Figure 7, Figures S29 and S30), while in predicted complexes **7** and **8**, residue Phe1307 of GluN2B-CT_1290-1310_ interacts with the hydrophobic pocket (Leu19, Val27, Leu95, and Met146) of DAPK1 (Figures S31 and S32). In addition, several other residues were identified in the DAPK1/GluN2B-CT_1290-1310_ interface of predicted complex **1** (Arg302, Glu100, Glu143, Arg1300, Arg1300 and Asp1305), complex **3** (Glu143, Asn144, Glu100, Arg1295, Asn1294, and Asp1309), complex **4** (Arg1299, Arg1300 and Asp1305), complex **5** (Gln23, Asp139, Asp161, Glu182, Arg1295 and Arg1300), complex **7** (Val96, Glu100, Arg1300, Asp1309), and complex **8** (Glu18, Leu19, Asp1305 and Asp1306). Thus, Table 4 provides the interactions of GluN2B-CT_1290–1310_/DAPK1 at an atomic level, which may facilitate the discovery of new drugs that target the interface of the GluN2B-CT_1290–1310_/DAPK1complex.

## 3. Materials and Methods

### 3.1. Structure Preparation

GluN2B-CT_1290-1310_ is the C-terminal catalytic domain of the GluN2B subunit formed by the linear sequence of amino acid (1290AQKKNRNKLRRQHSYD-TFVDL1310) [49]. First of all, program LEaP embedded in AMBER14 was applied to produce the linear starting structure of GluN2B-CT_1290-1310_ with the FF14SB force field [50]. Then, the prmtop and inpcrd files of the GluN2B-CT_1290-1310_ structure were created while the whole system was solvated in an octahedral periodic box of TIP3P water molecules [50]. The distance between the edge of the water box and the closest atom of solutes was at least 10 Å. To neutralize the charges of the system, an appropriate number of Cl^−^ counter ions were added. Finally, the prepared system was subjected to three independent (parallel), long-time (600 ns for each trajectory) MD simulations to predict the native structure of GluN2B-CT_1290-1310_. All MD simulations were fully unrestrained and carried out using the *SANDER* module [51], which was modified to improve its performance on the Linux/Intel PC cluster. Solvation effects were incorporated using the Generalized Born model, as implemented in AMBER.

The 2 Å resolution X-ray crystal structure of DAPK1 was obtained from the Protein Data Bank (PDB code 2XZS) [17]. The initial configuration of the free protein for the simulation was obtained by removing the protein B chain, Mg^2+^ ion, and water from the crystal structure. The missing amino acids (45–47) were added. Protein Preparation Wizard [52] was used to prepare protein structures, add hydrogen atoms, assign partial charges based on the OPLS-2005 force field, assign protonation states, and minimize the structure.

### 3.2. Protein–Peptide Docking

Protein–peptide docking was performed to analyze interactions between DAPK1 and GluN2B-CT_1290-1310_. In this work, the DAPK1 crystal structure (PDB code: 2XZS) and the three modeled GluN2B-CT_1290-1310_ structures were subjected to the three different types of docking software: GRAMM-X [36], ZDOCK [37], and SwarmDock [38]. GRAMM-X docking was used to perform a rigid-body procedure using Fast Fourier Transform (FFT) through applying smoothed Lennard–Jones potential, knowledge-based, and refinement stage scoring to discover the best surface match [53,54,55]. ZDOCK integrate the Pairwise Shape Complementarity (PSC) with desolvation and electrostatic function scoring using a Fast Fourier Transform algorithm [56,57,58]. SwarmDock was carried out by a flexible docking procedure with a Particle Swarm Optimization (PSO) algorithm [59,60,61,62]. For each docking, the top 10 docked poses were saved to select the initial binding mode.

### 3.3. Molecular Dynamics Simulations

The structures of DAPK1 and GluN2B-CT_1290-1310_ were modeled using LEaP embedded in AMBER14 with the standard AMBER ff14SB [50]. The system was neutralized and immersed into a rectangular periodic box of TIP3P [50] water molecules. Sufficient solvent was added to provide a minimum distance of 10 Å between any protein atoms and the edges of the box. MD simulations were carried out using the GPU (NVDIA Tesla K20C) accelerated PMEMD program in AMBER14. The simulations of system included the energy minimization, heating, equilibration and production run. First of all, energy minimization was carried out for the solvated complex in 2 steps, as follows: a harmonic restraint with a force constant of 2 kcal/(mol/Å^2^) was applied to all protein atoms and all atoms were allowed to move freely in turn. In each step, energy minimization was performed by the steepest descent method for the first 2500 steps and the conjugated gradient method for the subsequent 2500 steps. Then, the system heated up from 0 K to 300 K over 100 ps and equilibrated under a constant pressure of 1 atm and a constant temperature of 300 K for 5 ns. After the equilibration procedure, the production simulation was conducted for 200 ns at 300 K and 1 atm using periodic boundary conditions. For all simulations, long-range electrostatic interactions (cutoff = 10 Å) were calculated using the Particle Mesh Ewald (PME) algorithm [63], and bond lengths involving bonds to hydrogen atoms were constrained using the SHAKE algorithm [64].

### 3.4. Binding Free Energy Calculation and Per-Residue Energy Decomposition Analysis

For the simulated complex, 500 snapshots were extracted from the last 50 ns along the MD trajectory at intervals of 100 ps. The MM/GBSA [47,65,66,67,68,69,70,71,72,73,74,75,76,77] implemented in AMBER14 [78], and the electrostatic free energy of solvation (∆G_GB_) were calculated by solving GB equations and computing the binding free energies of the complex systems [79]. The binding energy (ΔG_GBTOT_) can be represented as follows:

(1) $$\;\Delta G_{\mathrm{GBTOT}}={\Delta E}_{\mathrm{vdW}}+{\Delta E}_{\mathrm{ELE}}+\Delta G_{\mathrm{GB}}+\Delta G_{\mathrm{GBSUR}}\;$$

where ΔG_GBTOT_ was obtained by summing the van der Waals (ΔE_vdW_) energies and the electrostatic energy (ΔE_ELE_) is the sum of polar (ΔG_GB_) and nonpolar (ΔG_GBSUR_) contributions. ∆E_vdW_ and ∆E_ELE_ were calculated using the AMBER ff14SB, and the electrostatic free energy of solvation (∆G_GB_) was calculated by solving the GB equation.

### 3.5. In-Silico Alanine Scanning Analysis

In-silico alanine scanning [76,80,81,82,83,84,85,86] was used to find the hotspot residues of interactions between DAPK1 and GluN2B-CT_1290-1310_. The whole process included the generation of mutated snapshots and the calculation of the binding free energy difference of the complex. Firstly, 500 snapshots were collected from the last 50 ns of the trajectory. Alanine mutation was generated by truncating the selected mutation residue at Cγ and replacing Cγ with a hydrogen atom at a distance of 1.09 Å from Cβ along the direction of Cγ-Cβ bond [80]. The topology files with alanine mutations were regenerated by the LEaP module in AMBER14,_ENREF_59_ENREF_48 and the MM/GBSA method [65,66] was used to calculate the relative binding free energy (ΔΔG), defined by difference between wild type (WT) and mutant (MUT) complexes, as shown below:

(2) $$\;\Delta \Delta G=\Delta G_{\mathrm{MUT}}-\Delta G_{\mathrm{WT}}\;$$

where ΔG_WT_ and ΔG_MUT_ refer to the binding free energies of the WT and MUT complexes, respectively.

### 3.6. Hydrogen Bond Analysis

To perform the hydrogen bond analysis, the CPPTRAJ module from the AMBER14 [78] was used to calculate the percentage of time that a hydrogen bond existed in the simulation trajectories. The formation of a hydrogen bond was defined as a distance of <3.50 Å between the donor and acceptor and a donor–hydrogen acceptor angle of >120°. The hydrogen bonds that existed more than 50% of the time were analyzed.

## 4. Conclusions

In this work, the construction of the GluN2B-CT_1290-1310_ structure, the docking of GluN2B-CT_1290-1310_ into the DAPK1 active site, and an MD simulation of the predicted GluN2B-CT_1290–1310_/DAPK1 docking complexes were performed to explore the interaction profile between GluN2B-CT_1290–1310_ and the DAPK1 interface. Based on the MD simulation, the per-residue free energy decomposition and an in-silico alanine scanning analysis, the interaction profile between GluN2B-CT_1290-1310_ and DAPK1 interface was identified. The results show that the residues Tyr1304 and Phe1307 interact with the hydrophobic pocket (Val27, Ile160, Leu95 and Met146) of DAPK1 and these hydrophobic interactions play crucial roles in DAPK1 and GluN2B-CT_1290-1310_ recognition. Notably, the concluded binding mode of the GluN2B-CT_1290-1310_/DAPK1 complex in this work is a pure computational modeling result and needs to be validated by experimental studies in the future.

## Figures and Tables

**Table 1:** Summary of the docking and MD simulations of the predicted GluN2B-CT1290-1310/DAPK1 complexes.

GluN2B-CT_1290-1310_/DAPK1 Complex	Structure of GluN2B-CT_1290-1310_	DAPK1 PDB Code *^a^*	Docking Program	Ranking Number in Docking *^b^*	Number of Water Molecules	Total Number of Atoms	Simulation Time
**1**	Trajectory A 50 ns	2XZS	GRAMM-X	2	15570	51941	200 ns
**2**	Trajectory A 600 ns	2XZS	GRAMM-X	5	12968	44135	200 ns
**3**	Trajectory C 600 ns	2XZS	GRAMM-X	4	11877	40862	200 ns
**4**	Trajectory A 600 ns	2XZS	ZDOCK	3	12013	41270	200 ns
**5**	Trajectory A 600 ns	2XZS	ZDOCK	9	12613	43070	200 ns
**6**	Trajectory C 600 ns	2XZS	ZDOCK	1	11897	40922	200 ns
**7**	Trajectory A 600 ns	2XZS	SwarmDock	1	12003	41240	200 ns
**8**	Trajectory B 600 ns	2XZS	SwarmDock	5	12748	43475	200 ns

*^a^*The 3D structure of DAPK1 from the Protein Data Bank (PDB) database (PDB code: 2XZS). *^b^*The ranking number of the selected complex from the 10 docking poses for further MD simulation.

**Table 2:** The binding free energies for predicted GluN2B-CT_1290–1310_/DAPK1 complex 1 (kcal/mol).

Energy Contribution	GluN2B-CT_1290–1310_/DAPK1		DAPK1		GluN2B		Delta	
	Mean	σ *^j^*	Mean	σ *^j^*	Mean	σ *^j^*	Mean	σ *^j^*
ELE *^a^*	−10147.50	108.22	−8860.67	108.82	−805.70	18.87	−481.09	34.11
VDW *^b^*	−1371.34	28.80	−1279.68	27.00	−17.06	7.32	−74.59	7.85
INT *^c^*	7827.43	54.97	7276.88	52.29	550.55	14.92	0.00	0.00
GAS *^d^*	−3691.36	119.20	−2863.47	115.65	−272.21	21.94	−555.68	35.79
GBSUR *^e^*	113.59	3.03	107.98	2.43	16.66	0.51	−11.05	1.10
GB *^f^*	−4397.01	98.49	−4284.63	99.70	−622.19	14.48	509.81	33.16
GBSOL*^g^*	−4283.42	96.91	−4176.65	98.54	−605.53	14.30	498.76	32.86
GBELE*^h^*	−14544.50	29.69	−13145.30	27.41	−1427.89	8.83	28.72	5.81
GBTOT *^i^*	−7974.78	53.64	−7040.12	50.22	−877.75	15.02	−56.92	8.73

^a^ Electrostatic energy as calculated by the molecular mechanics (MM) force field. ^b^ Van der Waals contribution. ^c^ Internal energy arising from bond, angle, and dihedral terms. ^d^ Total gas phase energy. ^e^ Non-polar contribution to the solvation free energy calculated by an empirical model. ^f^ The electrostatic contribution to the solvation free energy. ^g^ Sum of non-polar and polar contributions to solvation. ^h^ Sum of the electrostatic solvation free energy and molecular mechanics (MM) electrostatic energy. ^i^ Final estimated binding free energy calculated from the terms above. ^j^ Standard deviation.

**Table 3:** Analysis of hydrogen bond interactions between DAPK1 and GluN2B-CT_1290–1310_ complex **1**.

Acceptor	DonorH	Donor	Frames	Occupancy *^a^*	AvgDist *^b^*	AvgAng *^c^*
100@OE1	1300@HH11	1300@NH1	13754	68.77%	2.92	149.35
100@OE2	1300@HH11	1300@NH1	13009	65.04%	2.92	150.07
100@OE1	1300@HE	1300@NE	11175	55.87%	2.99	148.35
100@OE2	1300@HE	1300@NE	10038	50.19%	3.00	148.24
161@OD2	1302@HD1	1302@ND1	9403	47.02%	2.88	158.51
161@OD2	1303@HG	1303@OG	9091	45.46%	2.78	157.89
143@OE2	302@HH22	302@NH2	11794	58.97%	2.90	152.66
143@OE1	302@HH22	302@NH2	10289	51.44%	3.02	146.13
143@OE1	302@HH12	302@NH1	9107	45.53%	2.87	157.70
1298@O	302@HH11	302@NH1	13591	67.95%	2.86	157.30

*^a^*H-bond occupancy (%) as defined by the fraction of frames to evaluate the stability and the strength of the hydrogen bonds; only hydrogen bonds that existed more than 40% of the time were analyzed. *^b^*The hydrogen bonds were determined as those having a donor-acceptor distance of less than 3.50 Å. *^c^*Acceptor H-donor angle of greater than 120°.

**Table 4:** Summary of the identified interface residues shown to contribute to GluN2B-CT_1290–1310_/DAPK1 binding in the six analyzed simulation complexes.

GluN2B-CT_1290–1310_/DAPK1 Complex	Identified Interface Residues
**1**	Val27, Glu100, Glu143, Met146, Ile160, Asp161, Arg302, Asp1305, Tyr1304, His1302, Arg1300, Leu1298
**3**	Val27, Glu100, Glu143, Asn144, Met146, Asp161, Arg1295, Asp1309, His1302, Ser1303, Phe1307
**4**	Gln23, Val27, Glu143, Met146, Leu164, Phe178, Phe183, Arg1300, Asp1305, Arg1299, His1302, Tyr1304
**5**	Gln23, Glu143, Asp139, Asp161, Leu164, Thr180, Glu182, Arg1300, Arg1295, His1302, Ser1303, Tyr1304, Thr1306, Phe1307
**7**	Leu19, Val27, Val96, Ala97, Glu107, Met146, Lie160, Arg1295, Phe1307, Arg1309
**8**	Glu18, Leu19, Val27, Leu95, Asp103, Met146, Lys1297, Asp1305, Thr1306, Phe1307

## Supplementary Materials

The following are available online, Figures S1–S3: The representative structure extracted from the final 100 ns of the trajectory A, B and C MD simulation, Figures S4–S12: The docking poses of DAPK1 (PDB code 2XZS) and GluN2B-CT_1290-1310_ (GluN2B-CT_1290-1310_ was extracted from the final 100 ns MD trajectory A, B and C) were obtained from GRAMM-X, ZDOCK, SwarmDock, Figure S13: The docking poses of DAPK1 (PDB code 2XZS) and GluN2B-CT_1290-1310_ (GluN2B-CT_1290-1310_ was extracted from the first 50 ns MD trajectory A) were obtained from GRAMM-X, Figures S14–S19: The docking poses of DAPK1 (PDB code 1JKS and 5AV4) and GluN2B-CT_1290-1310_ (GluN2B-CT_1290-1310_ was extracted from the final 100 ns MD trajectory A, B and C) were obtained from GRAMM-X, Figure S20: The superimposition of DAPK1 crystal structures of 2XZS, 1JKS and 5AV4, Figure S21: The monitored RMSD of the all atoms with respect to the initial structure during the 200 ns simulation of GluN2B-CT_1290-1310_/DAPK1 complexes, Figure S22: The RMSF value of backbone atoms of GluN2B-CT_1290-1310_/DAPK1 complexes during the 200 ns MD simulation, Figures S23–S32: Per-residue binding energy decomposition, alanine scanning analyses and key interactions of the predicted complex 3, 4, 5, 7 and 8, Tables S1–S5: The binding free energies for the predicted complex 3, 4, 5, 7 and 8, Tables S6–S10: Analysis of hydrogen bond interactions between DAPK1 and GluN2B-CT_1290–1310_ complex 3, 4, 5, 7 and 8.
