# Supplementary material for: Prediction of GluN2B-CT1290-1310/DAPK1 Interaction by Protein–Peptide Docking and Molecular Dynamics Simulation
Source: Molecules. 2018 Nov 19;23(11):3018. doi: 10.3390/molecules23113018 (PMC6278559; doi:10.3390/molecules23113018)
Supplement: Supplementary file 1 [file molecules-23-03018-s001.pdf]

Supplementary Information for:

Prediction of GluN2B-CT$_{1290-1310}$/DAPK1 Interaction by Protein-peptide Docking and Molecular Dynamics Simulation

Gao TU[†,§], Tingting FU[†,§], Fengyuan YANG[†,§], Lixia YAO[ʃ], Weiwei XUE*[,†] and Feng ZHU*[,†,§]

[†] Innovative Drug Research and Bioinformatics Group, School of Pharmaceutical Sciences and Collaborative Innovation Center for Brain Science, Chongqing University, Chongqing 401331, China

[§] Innovative Drug Research and Bioinformatics Group, College of Pharmaceutical Sciences, Zhejiang University, Hangzhou 310058, China

[ʃ] Department of Health Sciences Research, Mayo Clinic, Rochester MN 55905, United States

Corresponding Author

* Feng ZHU (zhufeng@zju.edu.cn & prof.zhufeng@gmail.com), College of Pharmaceutical Sciences, Zhejiang University, Hangzhou 310058, China.Weiwei XUE (xueww@cqu.edu.cn), School of Pharmaceutical Sciences, Chongqing University, Chongqing 401331, China

Supplementary Results

The Modeled Structure of GluN2B C-terminal Peptide

For trajectory A, the representative snapshots of the GluN2B-$CT_{1290-1310}$ during the simulation were extracted and displayed in **Figure S1**. Compared with the initial conformation, the structure of GluN2B-$CT_{1290-1310}$ folded into a three-dimensional structure, and an internal hydrogen bonds network was formed among residues Lys1293, Asn1294, Lys1297, Arg1295, Asn1296, Asp1309, Asp1305 and Arg1300 (**Figure S1**). Moreover, the hydrogen bond between the side chains of Arg1300 and Asp1305, Arg1295 and Asp1309 may stabilize the folded states of the structure.

For trajectory B, the representative snapshots of the GluN2B-$CT_{1290-1310}$ during the final 100 ns MD simulation were extracted and displayed (**Figure S2**). Compared with the initial conformation, the structure of GluN2B-$CT_{1290-1310}$ folded into a three-dimensional structure, and an internal hydrogen bonds network was formed among residues Asn1294, Arg1295, Asn1297, Tyr1304, Asp1305, and Asp1309 (**Figure S2**). The structure of GluN2B-$CT_{1290-1310}$ folded into a three-dimensional structure due to the several internal hydrogen bonds between the backbone atoms may play important roles in stabilizing the folded state of GluN2B-CT1290-1310, and the crucial residues including Arg1295 and Asp1305, Arg1299 and Asp1309, Tyr1304 and Asn1297 (**Figure S2**).

As shown in **Figure S3**, an internal hydrogen bonds network was formed among residues Gln1291, Arg1295, Arg1299, His1302, Ser1303, Tyr1304, Thr1306 and Asp1309 in the representative structure of trajectory C, and several residues Arg1300 and Asp1309, Ser1303 and Tyr1304 may stabilized the folded states of the structure.

Molecular Dynamics Simulation of GluN2B-CT1290-1310/DAPK1 Complexes

As illustrated in **Figure** S21, the RMSDs underwent a small displacement 3~4 Å, indicating that simulation trajectories were converged during 200 ns MD simulation. Unfortunately, it can be observed that the RMSD value of the predicted complex 2 (**Figure S21A**) and complex 6 (**Figure S21E**) were fluctuate drastically during the simulation. In addition, the RMSF value of the complexes were calculated over the 200 ns to quantitatively monitor the fluctuation of each residue during the 200ns simulation (**Figure** S22). It is showed that the fluctuation for GluN2B-$CT_{1290-1310}$/DAPK1 complexes is nearly constant in the DAPK1. Especially the predicted complex 2 (**Figure S22A**) and complex 6 (**Figure S22E**) two systems, residues with higher value of RMSF displayed more flexible residues (residue 1290 to 1310) and indicating more movement observed along simulations of the predicted GluN2B-$CT_{1290-1310}$/DAPK1 complexes. Analysis of the representative structures shown that the peptide escape from the DAPK1 phosphorylation ATP binding pocket. It is proposed that due to the incorrect initial conformation of

complex 2 and 6 are unreasonable. Because this kind rare event would not be occurred in this classical MD simulation.

Calculation of GluN2B-CT$_{1290\text{-}1310}$/DAPK1 Complexes Binding Free Energy

In the predicted GluN2B-CT$_{1290\text{-}1310}$/DAPK1 complex 3 (**Table S1**), the major favorable contribution (-635.30 kcal/mol) to the protein-peptide was the electrostatic energy term (ΔE_{ELE}), the polar solvation energy (GB) to the solvation free energy term (ΔGB_{TOT}) was unfavorable (675.65 kcal/mol) to the interaction and the van der Waals energy (ΔE_{VDW}) for the predicted GluN2B-CT$_{1290\text{-}1310}$/DAPK1 complex is -83.40 kcal/mol, which was favorable contributions. In the predicted GluN2B-CT$_{1290\text{-}1310}$/DAPK1 complex 4 (**Table S2**), the major favorable contributor (-460.15 kcal/mol) to the protein-peptide was the electrostatic energy term (ΔE_{ELE}), the polar solvation energy (GB) to the solvation free energy term (ΔGB_{TOT}) was unfavorable (519.64 kcal/mol) to the interaction and van der Waals energy (ΔE_{VDW}) for the predicted GluN2B-CT$_{1290\text{-}1310}$/DAPK1 complex is -86.41 kcal/mol, which was favorable contributions. In the predicted GluN2B-CT$_{1290\text{-}1310}$/DAPK1 complex 5 (**Table S3**), the major favorable contributor (-427.51 kcal/mol) to the protein-peptide was the electrostatic energy term (ΔE_{ELE}), the polar solvation energy (GB) to the solvation free energy term (ΔGB_{TOT}) was unfavorable (474.25 kcal/mol) to the interaction. The van der Waals energy (ΔE_{VDW}) for the predicted GluN2B-CT$_{1290\text{-}1310}$/DAPK1 complex is -86.21 kcal/mol, which was favorable contributions. In the predicted GluN2B-CT$_{1290\text{-}1310}$/DAPK1 complex 7 (**Table S4**), the major favorable contributor (-592.21 kcal/mol) to the protein-peptide was the electrostatic energy term (ΔE_{ELE}), the polar solvation energy (GB) to the solvation free energy term (ΔGB_{TOT}) was unfavorable (641.12 kcal/mol) to the interaction. The van der Waals energy (ΔE_{VDW}) for the predicted GluN2B-CT$_{1290\text{-}1310}$/DAPK1 complex is -81.28 kcal/mol, which was favorable contributions. In the predicted GluN2B-CT$_{1290\text{-}1310}$/DAPK1 complex 8 (**Table S5**), the major favorable contributor (-606.89 kcal/mol) to the protein-peptide was the electrostatic energy term (ΔE_{ELE}), whereas the polar solvation energy (GB) to the solvation free energy term (ΔGB_{TOT}) was unfavorable (634.30 kcal/mol) to the interaction. The van der Waals energy (ΔE_{VDW}) for the predicted GluN2B-CT$_{1290\text{-}1310}$/DAPK1 complex is -57.40 kcal/mol, which was favorable contributions.

Free Energy Decomposition Analysis of GluN2B-CT$_{1290\text{-}1310}$/DAPK1 Complexes

As can be seen in **Figure** S23**A,** the major energy contribution for the predicted complex 3 was from the residues Asn144, Asp161, Leu164, Lys1292, Asn1294, Arg1295, Arg1299, His1302, Phe1307, Asp1309, Leu1310 were more than 2 kcal/mol. Especially for residues Arg1295, Lys1292 and Phe1307, the corresponding absolute energy contribution residues are -6.51, -5.61 and -4.67 kcal/mol, respectively. In the predicted GluN2B-CT$_{1290\text{-}1310}$/DAPK1 complex 4, the contribution of 28 major residues (more than 0.60 kcal/mol) of the complex were compared and plotted in **Figure** S24**A**. The absolute energy contribution residues Gln23, Glu143, Phe178, Gln1301 and Tyr1304 was more than 2 kcal/mol. Especially

for residues Gln23, Phe178, Tyr1304, the absolute energy contribution residues are -4.01, -5.25 and -3.66 kcal/mol, respectively. In the predicted GluN2B-$CT_{1290-1310}$/DAPK1 complex 5, the contribution of 26 major residues (more than 0.60 kcal/mol) of the complex, were compared and plotted in **Figure S25A**. The absolute energy contribution residues Gln23, Asp139, Leu164, Glu182, Arg1295, Arg1300, His3102, Ser1303, Tyr1304, Thr1306, Phe1307 and Leu1310 were more than 2 kcal/mol. Especially for residues Asp139 and Tyr1304, the absolute energy contribution residues are -11.93, -6.09 kcal/mol, respectively. In the predicted GluN2B-$CT_{1290-1310}$/DAPK1 complex 7, the contribution of 27 major residues (more than 0.60 kcal/mol) of the complex, were displayed in **Figure S26A**. The absolute energy contribution residues Leu19, Glu107, Asn151, Arg1295, Phe1307, Val1308 and Leu1310 were more than 2 kcal/mol. Especially for residues Arg1295, Phe1307, the corresponding energy contribution are -4.70 and -6.01 kcal/mol, respectively. In the predicted GluN2B-$CT_{1290-1310}$/DAPK1 complex 8, the contribution of 18 major residues (more than 0.60 kcal/mol) of the complex, were compared and plotted in **Figure S27A**. The absolute energy contribution residues Glu18, Leu19, Asp103, Glu107, Arg1295, Lys1297, Arg1299, Phe1307 and Leu1310 were more than 2 kcal/mol. Especially for residues Leu19, Lys1297 and Phe1307, the corresponding absolute energy contribution are -4.02, -6.68 and -6.54 kcal/mol, respectively.

In Silico Alanine Scanning Analysis of GluN2B-$CT_{1290-1310}$/DAPK1 Complexes

For the predicted GluN2B-$CT_{1290-1310}$ /DAPK1 complex 3, **Figure S23B** shown the results of selected 11 interface residues (Asn144, Asp161, Leu164, Lys1292, Asn1294, Arg1295, Arg1299, His1302, Phe1307, Asp1309 and Leu1310) by *in silico* alanine scanning analysis and the results clearly indicates that the selected 11 residues were important for the interaction of DAPK1 and GluN2B-$CT_{1290-1310}$. In the predicted GluN2B-$CT_{1290-1310}$/DAPK1 complex 4, the results of *in silico* alanine scanning analysis for selected 5 residues in GluN2B-$CT_{1290-1310}$ /DAPK1 interface were displayed in **Figure S24B**, it can be observed that the 5 residues (Gln23, Glu143, Phe178, Gln1301 and Tyr1304) contribute significantly (|contribution| ≥2 kcal/mol) to the binding of DAPK1 and GluN2B-$CT_{1290-1310}$. In the predicted GluN2B-$CT_{1290-1310}$/DAPK1 complex 5, the results of i*n silico* alanine scanning analysis for selected 12 residues in GluN2B-$CT_{1290-1310}$ /DAPK1 interface were shown in **Figure S25B**, residues (Gln23, Asp139, Leu164, Glu182, Arg1295, Arg1300, His3102, Ser1303, Tyr1304, Thr1306, Phe1307 and Leu1310) contribute significantly (|contribution| ≥2 kcal/mol) to the binding of DAPK1 and GluN2B-$CT_{1290-1310}$. In the predicted GluN2B-$CT_{1290-1310}$/DAPK1 complex 7, the results of *in silico* alanine scanning analysis for selected 7 residues in GluN2B-$CT_{1290-1310}$ /DAPK1 interface were summarized in **Figure S26B**, 6 residues (Leu19, Glu107, Asn151, Arg1295, Phe1307 and Leu1310) contribute significantly (|contribution| ≥2 kcal/mol) to the binding of DAPK1 and GluN2B-$CT_{1290-1310}$. In the predicted GluN2B-$CT_{1290-1310}$/DAPK1 complex 8, the results of in silico alanine scanning analysis for selected 9 residues in GluN2B-$CT_{1290-1310}$ /DAPK1 interface were summarized in **Figure S27B**, 9 residues (Glu18, Leu19, Asp103, Glu107, Arg1295, and

Lys1297, Arg1299, Phe1307 and Leu1310) contribute significantly (|contribution| ≥2 kcal/mol) to the binding of DAPK1 and GluN2B-$CT_{1290-1310}$.

Hydrogen Bond Interactions Network Analysis of GluN2B-$CT_{1290-1310}$/DAPK1 Complexes

For the predicted GluN2B-$CT_{1290-1310}$/DAPK1 complex 3, the persistence of identified hydrogen bond along the time of MD simulation was summarized in **Table S6**, residues Glu143, Asp1309 interacted with residue Arg1295 forms hydrogen bond, occupying more than 50% during the MD simulations. In addition, the residues His1303 and Asp161, residues Glu100 and Asn1294 form hydrogen bond (**Figure S**28). For the predicted GluN2B-$CT_{1290-1310}$/DAPK1 complex 4, the persistence of identified hydrogen bond along the time of MD simulation was summarized in **Table S7**. Residues His1302 and Tyr1304 close to the phosphorylation ATP binding pocket. Residue Arg1299 forms hydrogen bond with backbone carbonyl oxygen atom of Phe178. For the peptide GluN2B-$CT_{1290-1310}$, a salt bridge formed between Arg1300 and Asp1305 may typically stabilizes the peptide main conformation (**Figure S**29**).** Residues Gln23 has engaged in hydrogen bond with the backbone nitrogen of Asp1305 in GluN2B-$CT_{1290-1310}$/DAPK1, existing for more than 50% of the duration of the simulation. For the predicted GluN2B-$CT_{1290-1310}$/DAPK1 complex 5, a strong electrostatic attraction between Arg1300 and Asp139 **(Figure** S30). The peptide residues Tyr1304 and His1302 inserted into the phosphorylated site regions and the phosphorylation site Ser1303 forms hydrogen bond with the backbone nitrogen hydrogen atom of Gln23. Besides, Tyr1304 forms hydrogen bonds with backbone carbonyl oxygen atom of Asp161, residues Arg1295 has engaged in hydrogen bond with Glu182 in GluN2B-$CT_{1290-1310}$/DAPK1. From these simulation results, we concluded that the interface residues His1302, Tyr1304 close to the phosphorylation ATP binding pocket and play crucial roles to the binding mode of DAPK1/GluN2B-$CT_{1290-1310}$ interactions. The persistence of identified hydrogen bond was summarized in **Table S8**. It can be seen that a strong electrostatic attraction with average distance 3 Å between Arg1300 and Asp139, occupying more than 65% during the MD simulations. For the predicted GluN2B-$CT_{1290-1310}$/DAPK1 complex 7, it can be seen that a strong electrostatic attraction with average distance less than 3 Å between Arg1300 and Glu100, occupying more than 50% during the MD simulations (**Table S9**), the backbone nitrogen atom of Asp1309 forms hydrogen bonds with backbone carbonyl oxygen atom of Val97 in GluN2B-$CT_{1290-1310}$/DAPK1. The residues phe1307 of the peptide towards into the catalytic domain of DAPK1. The hydrophobic residues (Ieu19, Val27, Leu95, Val96 and Met146 and Ile160) that constitutes a hydrophobic pocket on the surface of DAPK1 interacted with the Phe307 of peptide GluN2B-$CT_{1290–1310}$ via hydrophobic interactions (**Figure S**31). For the predicted GluN2B-$CT_{1290-1310}$/DAPK1 complex 8, hydrogen bonds occur between the backbone oxygen of residues Gln18 and Leu19 with the backbone hydrogen atom of the residues Thr1306 and Asp1305 (**Table S10**). And the hydrophobic interaction between residues phe1307 and the hydrophobic pocket (Ieu19, Val27, Leu95 and Met146) of protein DAPK1, which may contribute to the

interaction of GluN2B-$CT_{1290\text{-}1310}$/DAPK1 (**Figure** S32). Based on the simulation results, we conclude that hydrophobic pocket (Ieu19, Val27, Leu95, Val96 and Met146 and Ile160) at the DAPK1 interface are also essential for the binding interactions. Therefore, electrostatic interaction and van der Waals interaction played the major role in the binding of GluN2B-$CT_{1290\text{-}1310}$/DAPK1. The hydrophobic pocket also contributes to the interaction of the complex.

Supplementary Tables

Table S1. The binding free energies for the predicted GluN2B-CT$_{1290–1310}$/DAPK1 complex 3 (kcal/mol)

Energy contribution	GluN2B-CT$_{1290–1310}$/DAPK1		DAPK1		GluN2B		Delta	
	Mean	σ	Mean	σ	Mean	σ	Mean	σ
ELE	-10116.80	125.12	-8713.32	125.54	-768.21	20.34	-635.30	38.86
VDW	-1372.44	28.41	-1256.56	27.80	-32.48	6.98	-83.40	6.34
INT	7789.04	52.17	7240.24	50.34	548.81	14.31	0.00	0.00
GAS	-3700.22	133.43	-2729.64	130.72	-251.88	23.61	-718.70	39.93
GBSUR	112.17	1.97	109.61	1.98	15.41	0.46	-12.86	0.71
GB	-4412.63	111.30	-4428.25	109.74	-660.03	16.85	675.65	37.08
GBSOL	-4300.46	110.35	-4318.64	108.85	-644.62	16.69	662.79	36.80
GBELE	-14529.50	33.05	-13141.60	32.88	-1428.24	7.92	40.35	7.94
GBTOT	-8000.69	53.32	-7048.28	51.31	-896.50	14.60	-55.91	7.10

Table S2. The binding free energies for the predicted GluN2B-CT$_{1290–1310}$/DAPK1 complex 4 (kcal/mol)

Energy contribution	GluN2B-CT$_{1290–1310}$/DAPK1		DAPK1		GluN2B		Delta	
	Mean	σ	Mean	σ	Mean	σ	Mean	σ
ELE	-10052.20	119.70	-8784.45	115.91	-807.61	20.63	-460.15	43.87
VDW	-1390.16	30.16	-1265.04	27.66	-38.71	6.85	-86.41	7.15
INT	7823.69	52.71	7283.09	50.96	540.60	14.49	0.00	0.00
GAS	-3618.67	126.03	-2766.41	120.36	-305.71	24.20	-546.55	45.11
GBSUR	111.19	2.20	108.93	1.79	14.73	0.38	-12.47	0.91
GB	-4434.90	113.40	-4353.71	108.67	-600.83	17.39	519.64	42.92
GBSOL	-4323.71	112.37	-4244.78	107.88	-586.11	17.22	507.17	42.51
GBELE	-14487.10	29.84	-13138.20	29.77	-1408.44	7.74	59.49	7.61
GBTOT	-7942.38	52.81	-7011.19	51.28	-891.82	14.73	-39.38	6.67

Table S3. The binding free energies for the predicted GluN2B-CT$_{1290–1310}$/DAPK1 complex 5 (kcal/mol)

Energy contribution	GluN2B-CT$_{1290–1310}$/DAPK1		DAPK1		GluN2B		Delta	
	Mean	σ	Mean	σ	Mean	σ	Mean	σ
ELE	-10133.30	131.23	-8847.82	108.76	-857.93	22.44	-427.51	86.58
VDW	-1383.91	28.50	-1277.85	26.32	-19.84	8.14	-86.21	5.65
INT	7784.50	53.94	7238.95	51.84	545.55	14.63	0.00	0.00
GAS	-3732.67	138.92	-2886.71	114.73	-332.23	26.78	-513.73	85.80
GBSUR	112.63	2.24	109.17	1.83	16.66	0.67	-13.19	0.70
GB	-4394.33	120.56	-4299.18	96.65	-569.40	17.46	474.25	80.09
GBSOL	-4281.69	119.19	-4190.01	95.95	-552.74	17.30	461.06	79.94
GBELE	-14527.60	31.20	-13147.00	29.41	-1427.33	9.50	46.74	10.25
GBTOT	-8014.36	55.52	-7076.72	53.28	-884.97	15.33	-52.67	8.55

Table S4. The binding free energies for the predicted GluN2B-$CT_{1290–1310}$/DAPK1 complex 7 (kcal/mol)

Energy contribution	GluN2B-$CT_{1290–1310}$/DAPK1		DAPK1		GluN2B		Delta	
	Mean	σ	Mean	σ	Mean	σ	Mean	σ
ELE	-10319.90	123.11	-8931.94	121.93	-795.78	29.34	-592.21	51.71
VDW	-1369.12	29.87	-1270.75	27.68	-17.09	6.92	-81.28	7.09
INT	7769.42	53.42	7230.86	51.18	538.56	14.08	0.00	0.00
GAS	-3919.64	132.79	-2971.83	131.22	-274.32	30.79	-673.49	53.84
GBSUR	113.20	2.27	106.72	1.86	17.64	0.54	-11.16	1.08
GB	-4197.71	111.84	-4215.97	111.42	-622.87	26.53	641.12	51.08
GBSOL	-4084.51	110.63	-4109.25	110.53	-605.23	26.31	629.96	50.45
GBELE	-14517.70	32.30	-13147.90	29.51	-1418.65	7.53	48.91	7.93
GBTOT	-8004.15	54.54	-7081.08	52.49	-879.54	14.03	-43.53	6.90

Table S5. The binding free energies for the predicted GluN2B-$CT_{1290–1310}$/DAPK1 complex 8 (kcal/mol)

Energy contribution	GluN2B-$CT_{1290–1310}$/DAPK1		DAPK1		GluN2B		Delta	
	Mean	σ	Mean	σ	Mean	σ	Mean	σ
ELE	-10051.40	113.75	-8759.23	99.63	-685.30	37.60	-606.89	32.17
VDW	-1396.46	29.14	-1298.31	27.39	-40.76	6.92	-57.40	5.80
INT	7830.36	54.69	7277.89	51.70	552.47	14.41	0.00	0.00
GAS	-3617.52	122.73	-2779.64	108.10	-173.59	36.59	-664.28	32.83
GBSUR	112.16	2.18	104.91	1.84	15.79	0.49	-8.54	0.65
GB	-4468.21	99.61	-4379.70	87.28	-722.82	34.86	634.30	30.63
GBSOL	-4356.05	98.72	-4274.79	86.74	-707.03	34.90	625.76	30.41
GBELE	-14519.60	32.37	-13138.90	30.51	-1408.12	7.90	27.42	6.17
GBTOT	-7973.57	55.79	-7054.43	52.74	-880.62	14.07	-38.52	6.52

Table S6. Analysis of hydrogen bond interactions between DAPK1 and GluN2B-$CT_{1290–1310}$ complex 3

Acceptor	DonorH	Donor	Frames	Occupancy[a]	AvgDist[b]	AvgAng[c]
161@OD2	1303@HG	1303@OG	18897	94.48%	2.63	163.14
143@OE2	1295@HH11	1295@NH1	12909	64.54%	2.90	152.78
143@OE1	1295@HH11	1295@NH1	12294	61.47%	2.94	150.95
100@OE1	1294@HD22	1294@ND2	10061	50.30%	2.92	158.61
143@OE1	1295@HE	1295@NE	8895	44.47%	3.01	153.40
1309@O	144@HD21	144@ND2	11446	57.23%	3.02	158.86
1309@OD1	1295@HH22	1295@NH2	11815	59.07%	3.05	142.21
1309@OD2	1295@HH12	1295@NH1	11572	57.86%	3.07	141.61

[a] H-bond occupancy (%) as defined by the fraction of frames, to evaluate the stability and the strength of the hydrogen bond and only hydrogen bonds that existed more than 40% of the time were analyzed.

[b] The hydrogen bonds are determined by donor-acceptor distance of less than 3.50 Å.

[c] The acceptor H-donor angle of greater than 120°.

Table S7. Analysis of hydrogen bond interactions between DAPK1 and GluN2B-CT$_{1290–1310}$ complex 4

Acceptor	DonorH	Donor	Frames	Occupancy[a]	AvgDist[b]	AvgAng[c]
161@HB2	1302@H	1302@N	10703	53.52%	3.00	142.34
23@OE1	1305@H	1305@N	10612	53.06%	2.96	148.33
178@O	1299@HH12	1299@NH1	8484	42.42%	2.88	153.33
1305@OD2	1300@HH11	1300@NH1	13032	65.16%	2.96	151.58
1305@OD1	1300@HH11	1300@NH1	12128	60.64%	3.03	145.72
1305@OD1	1300@HE	1300@NE	11114	55.57%	2.88	156.35

[a] H-bond occupancy (%) as defined by the fraction of frames, to evaluate the stability and the strength of the hydrogen bond and only hydrogen bonds that existed more than 40% of the time were analyzed.

[b] The hydrogen bonds are determined by donor-acceptor distance of less than 3.50 Å.

[c] The acceptor H-donor angle of greater than 120°.

Table S8. Analysis of hydrogen bond interactions between DAPK1 and GluN2B-CT$_{1290–1310}$ complex 5

Acceptor	DonorH	Donor	Frames	Occupancy[a]	AvgDist[b]	AvgAng[c]
161@O	1304@HH	1304@OH	19115	95.57%	2.80	161.71
143@OE2	1305@H	1305@N	17931	89.65%	2.94	154.42
143@OE1	1306@H	1306@N	16564	82.82%	2.96	149.58
139@OD2	1300@HH22	1300@NH2	15364	76.82%	2.99	148.16
139@OD1	1300@HH22	1300@NH2	13471	67.35%	3.09	148.93
143@OE1	1307@H	1307@N	12280	61.40%	3.01	164.08
182@OE2	1295@HH11	1295@NH1	11786	58.93%	2.83	154.31
161@O	1304@HE1	1304@CE1	8801	44.00%	3.32	128.52
182@OE1	1295@HH11	1295@NH1	8514	42.57%	2.90	151.48
1303@OG	23@H	20@N	14652	73.26%	3.02	155.43

[a] H-bond occupancy (%) as defined by the fraction of frames, to evaluate the stability and the strength of the hydrogen bond and only hydrogen bonds that existed more than 40% of the time were analyzed.

[b] The hydrogen bonds are determined by donor-acceptor distance of less than 3.50 Å.

[c] The acceptor H-donor angle of greater than 120°.

Table S9. Analysis of hydrogen bond interactions between DAPK1 and GluN2B-CT$_{1290–1310}$ complex 7

Acceptor	DonorH	Donor	Frames	Occupancy[a]	AvgDist[b]	AvgAng[c]
100@OE2	1300@HH12	1300@NH1	11002	55.01%	2.93	147.87
100@OE1	1300@HH12	1300@NH1	10923	54.61%	2.95	148.60
97@O	1309@H	1309@N	10835	54.17%	3.01	149.96
103@OD1	1295@HH22	1295@NH2	9525	47.62%	2.88	151.94
103@OD2	1295@HH12	1295@NH1	8013	40.06%	2.90	152.83

[a] H-bond occupancy (%) as defined by the fraction of frames, to evaluate the stability and the strength of the hydrogen bond and only hydrogen bonds that existed more than 40% of the time were analyzed.

[b] The hydrogen bonds are determined by donor-acceptor distance of less than 3.50 Å.

[c] The acceptor H-donor angle of greater than 120°.

Table S10. Analysis of hydrogen bond interactions between DAPK1 and GluN2B-CT$_{1290–1310}$ complex 8

Acceptor	DonorH	Donor	Frames	Occupancy[a]	AvgDist[b]	AvgAng[c]
18@O	1305@HA	1305@CA	10531	52.65%	3.31	132.07
19@O	1306@HA	1306@CA	9782	48.91%	3.34	145.41

[a] H-bond occupancy (%) as defined by the fraction of frames, to evaluate the stability and the strength of the hydrogen bond and only hydrogen bonds that existed more than 40% of the time were analyzed.

[b] The hydrogen bonds are determined by donor-acceptor distance of less than 3.50 Å.

[c] The acceptor H-donor angle of greater than 120°

Supplementary Figures

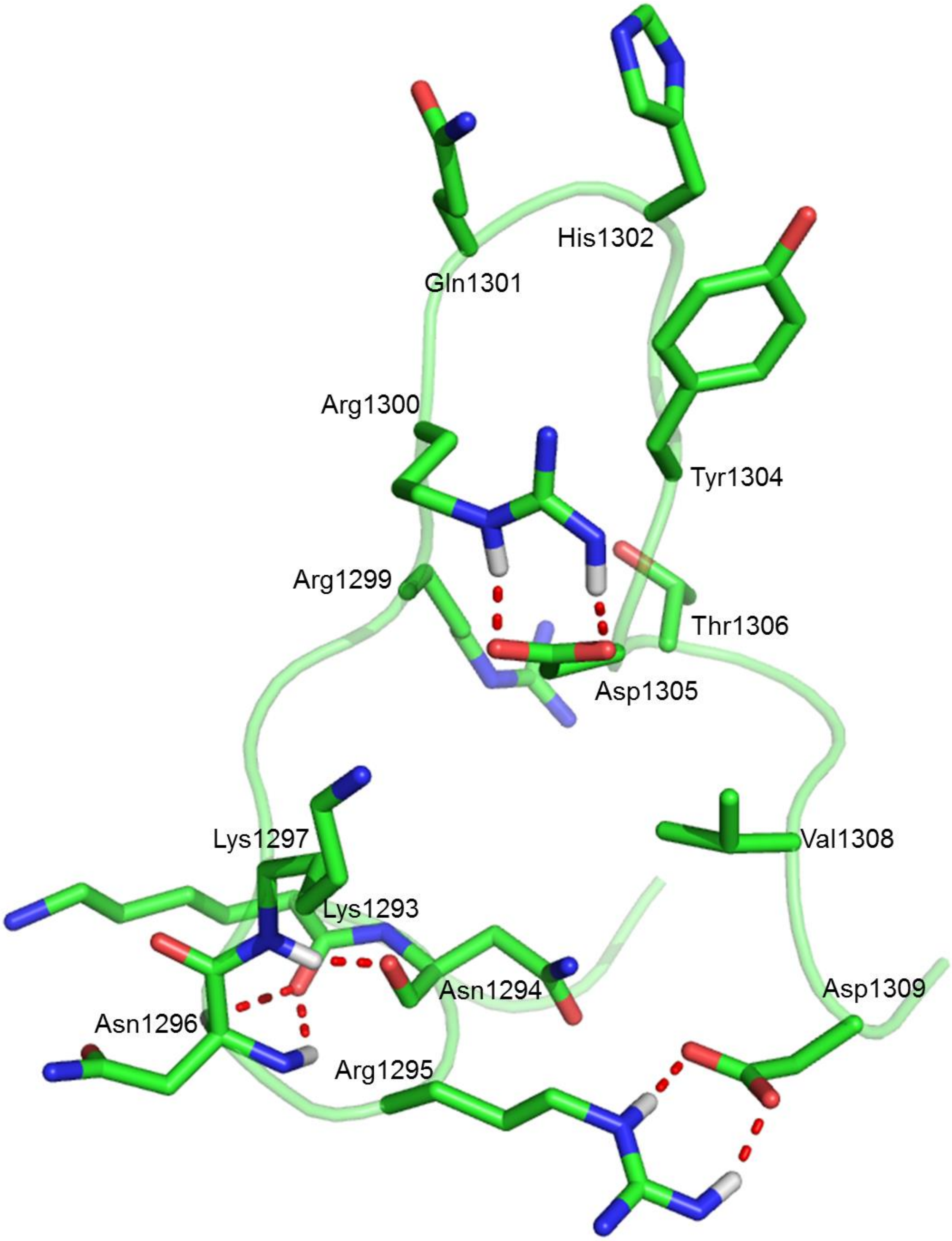

Figure S1. The representative structure extracted from the final 100 ns of the trajectory A MD simulation. The residues of the GluN2B-CT$_{1290–1310}$ are shown as green sticks. And hydrogen bond interactions are represented as red dashed lines.

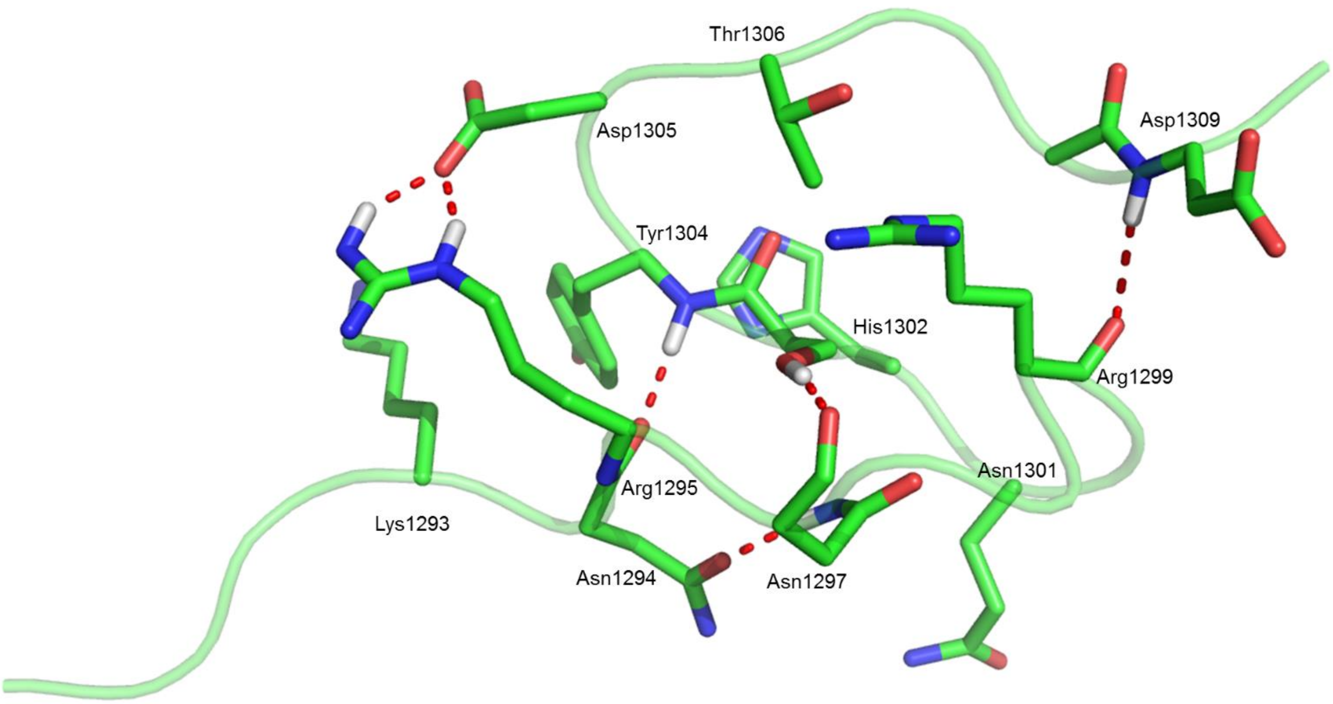

Figure S2. The representative structure extracted from the final 100 ns of the trajectory B MD simulation. The residues of the GluN2B-$CT_{1290–1310}$ are shown as green sticks. And hydrogen bond interactions are represented as red dashed lines.

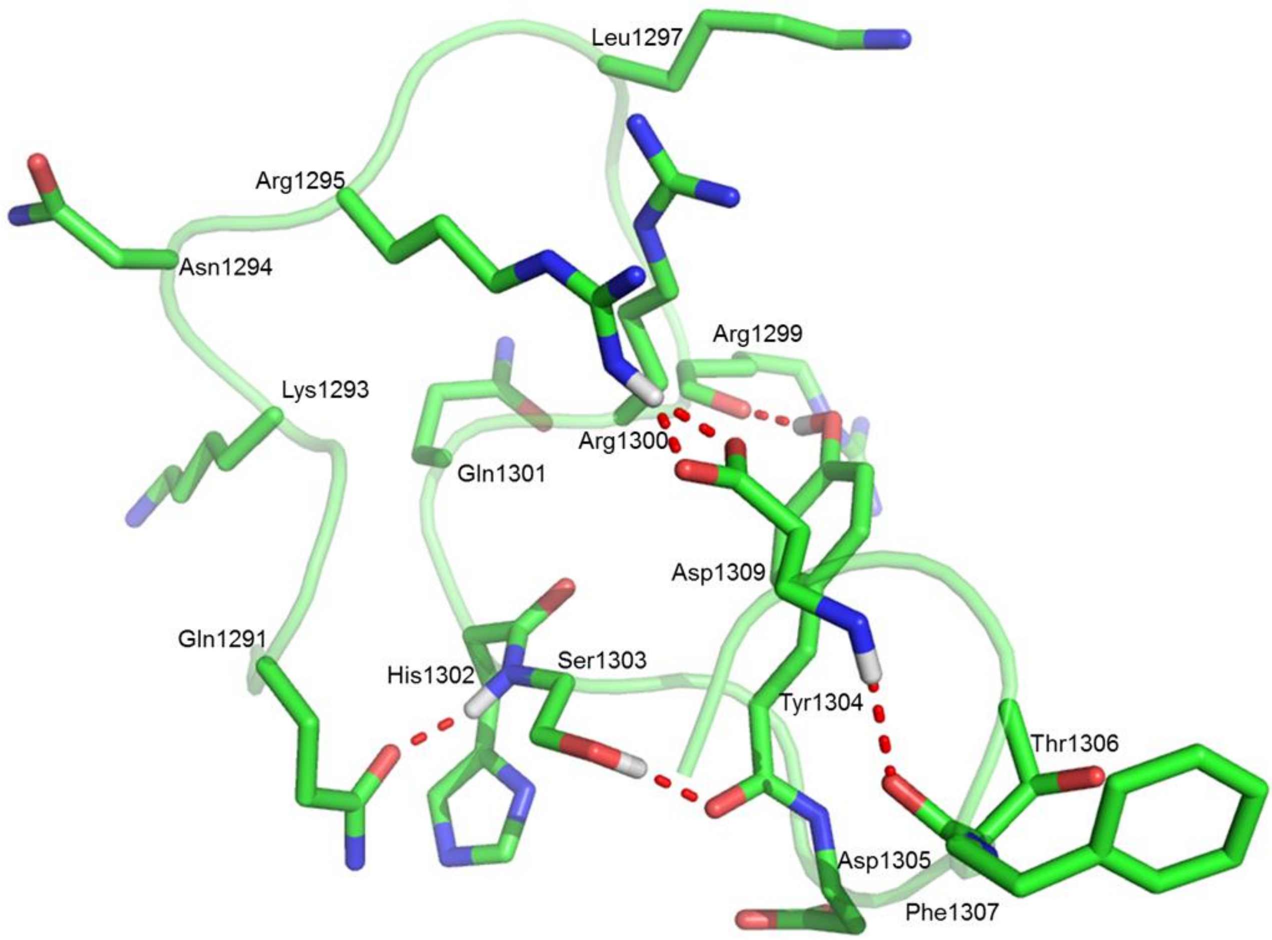

Figure S3. The representative structure extracted from the final 100 ns of the trajectory C MD simulation. The residues of the GluN2B-CT$_{1290–1310}$ are shown as green sticks. And hydrogen bond interactions are represented as red dashed lines.

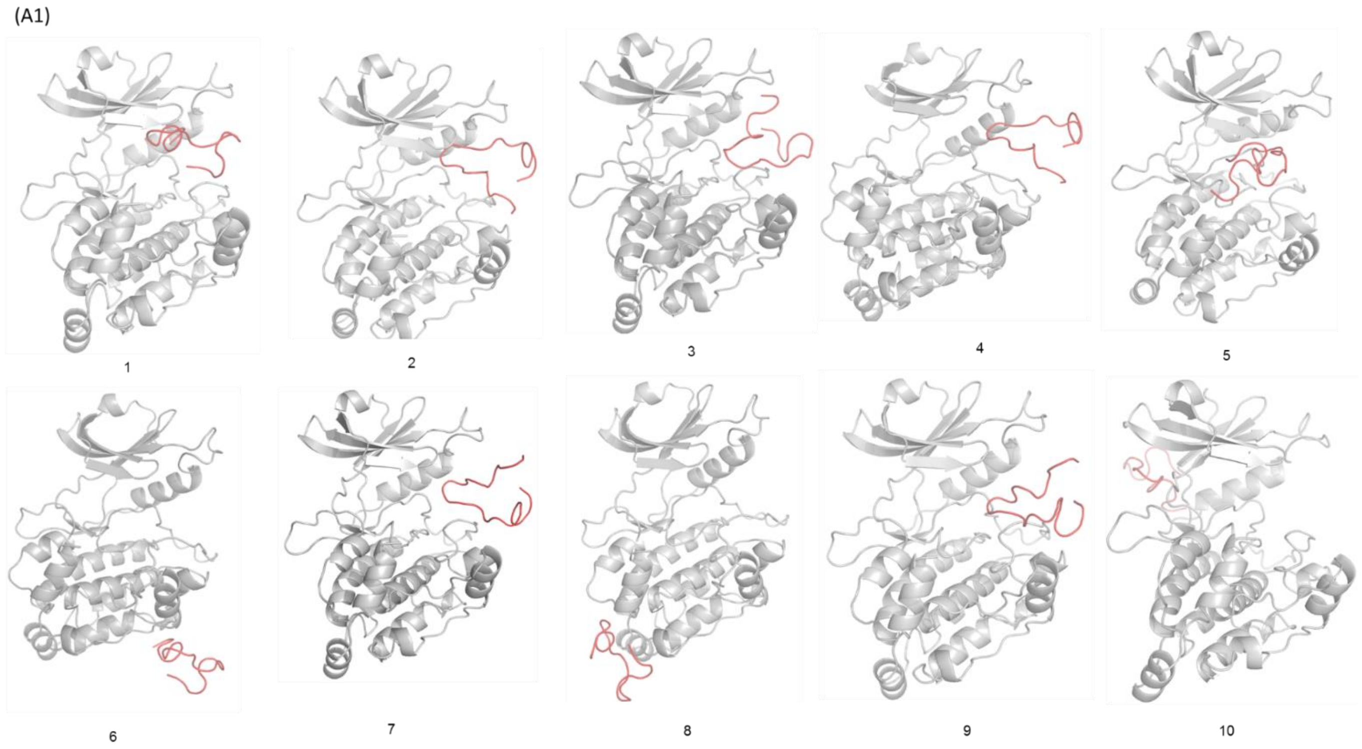

Figure S4. The docking poses of DAPK1 (PDB code 2XZS) and GluN2B-$CT_{1290-1310}$ were obtained from GRAMM-X. GluN2B-$CT_{1290-1310}$ was extracted from the final 100 ns MD trajectory A.

(B1)

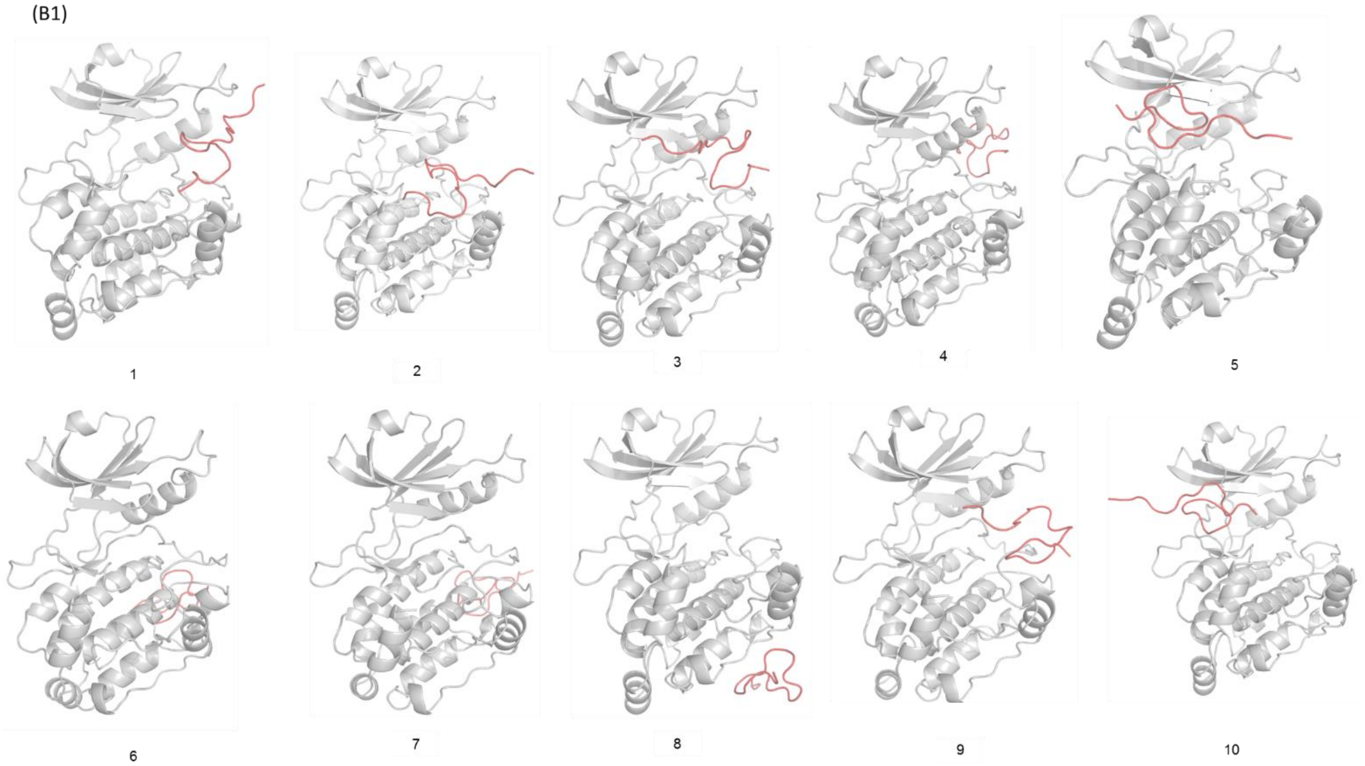

Figure S5. The docking poses of DAPK1 (PDB code 2XZS) and GluN2B-CT$_{1290-1310}$ were obtained from GRAMM-X. GluN2B-CT$_{1290-1310}$ was extracted from the final 100 ns MD trajectory B.

(C1)

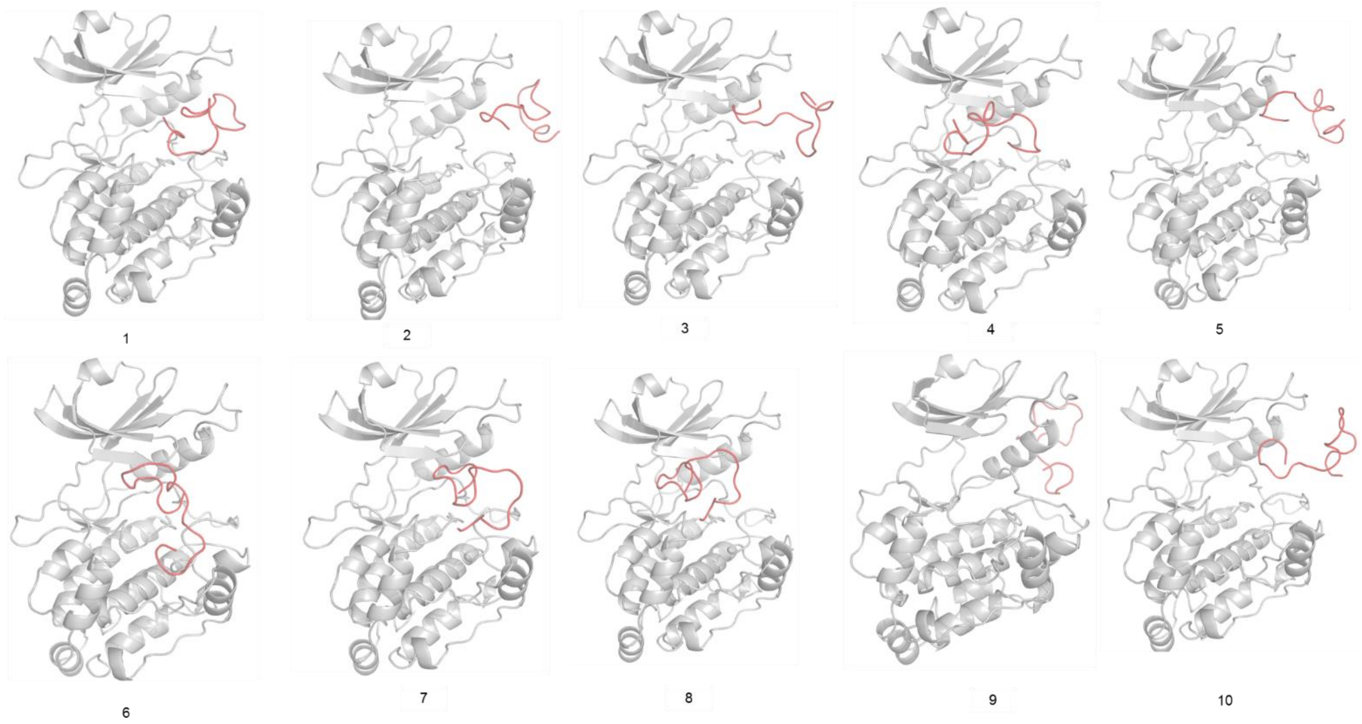

Figure S6. The docking poses of DAPK1 (PDB code 2XZS) and GluN2B-CT$_{1290\text{-}1310}$ were obtained from GRAMM-X. GluN2B-CT$_{1290\text{-}1310}$ was extracted from the final 100 ns MD trajectory C.

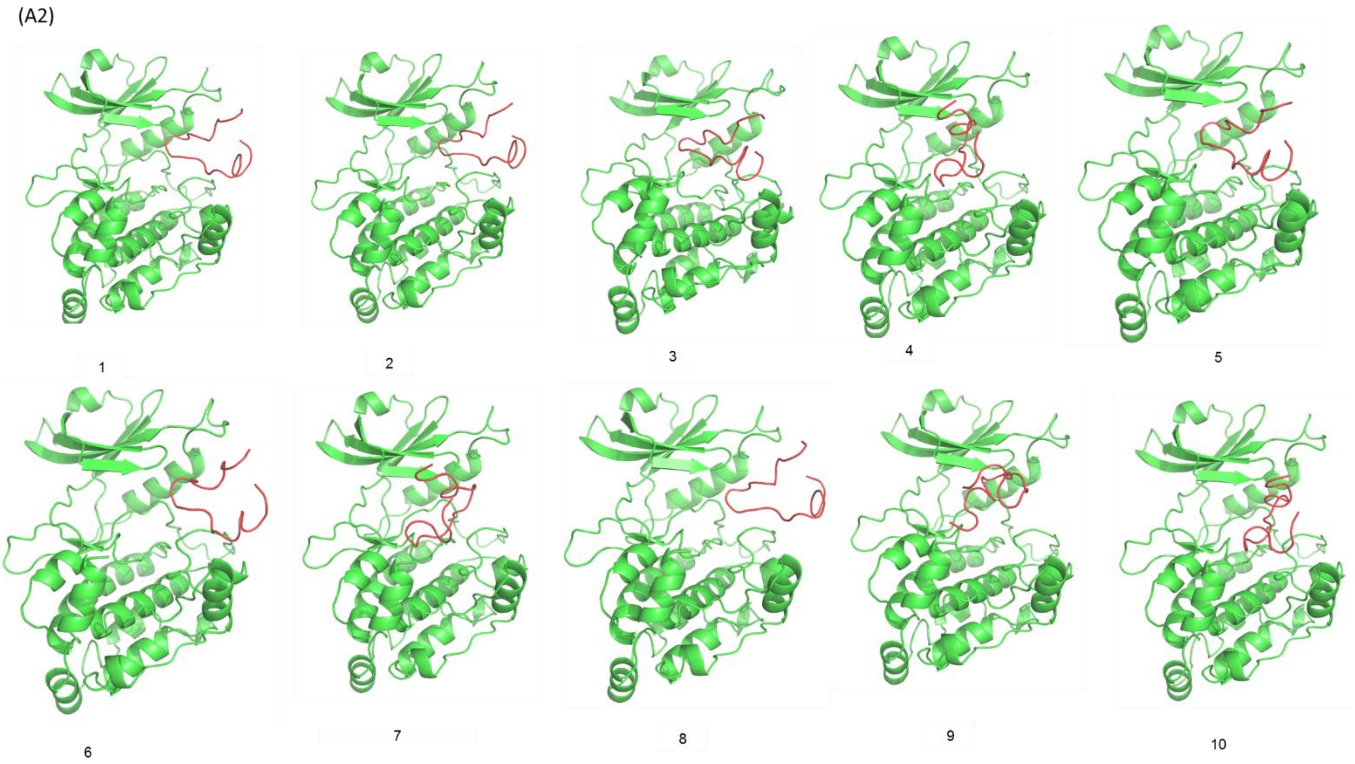

Figure S7. The docking poses of DAPK1 (PDB code 2XZS) and GluN2B-$CT_{1290\text{-}1310}$ were obtained from ZDOCK. GluN2B-$CT_{1290\text{-}1310}$ was extracted from the final 100 ns MD trajectory A.

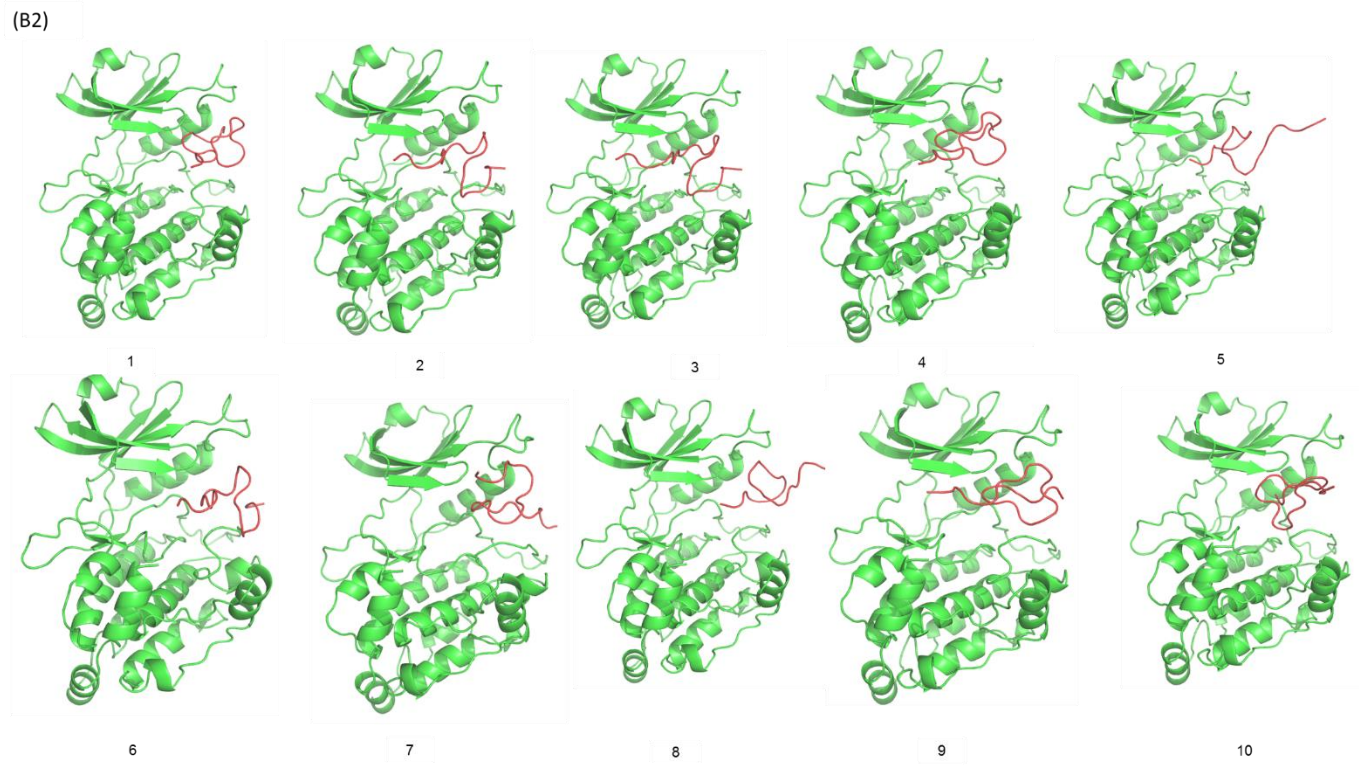

Figure S8. The docking poses of DAPK1 (PDB code 2XZS) and GluN2B-CT$_{1290\text{-}1310}$ were obtained from ZDOCK. GluN2B-CT$_{1290\text{-}1310}$ was extracted from the final 100 ns MD trajectory B.

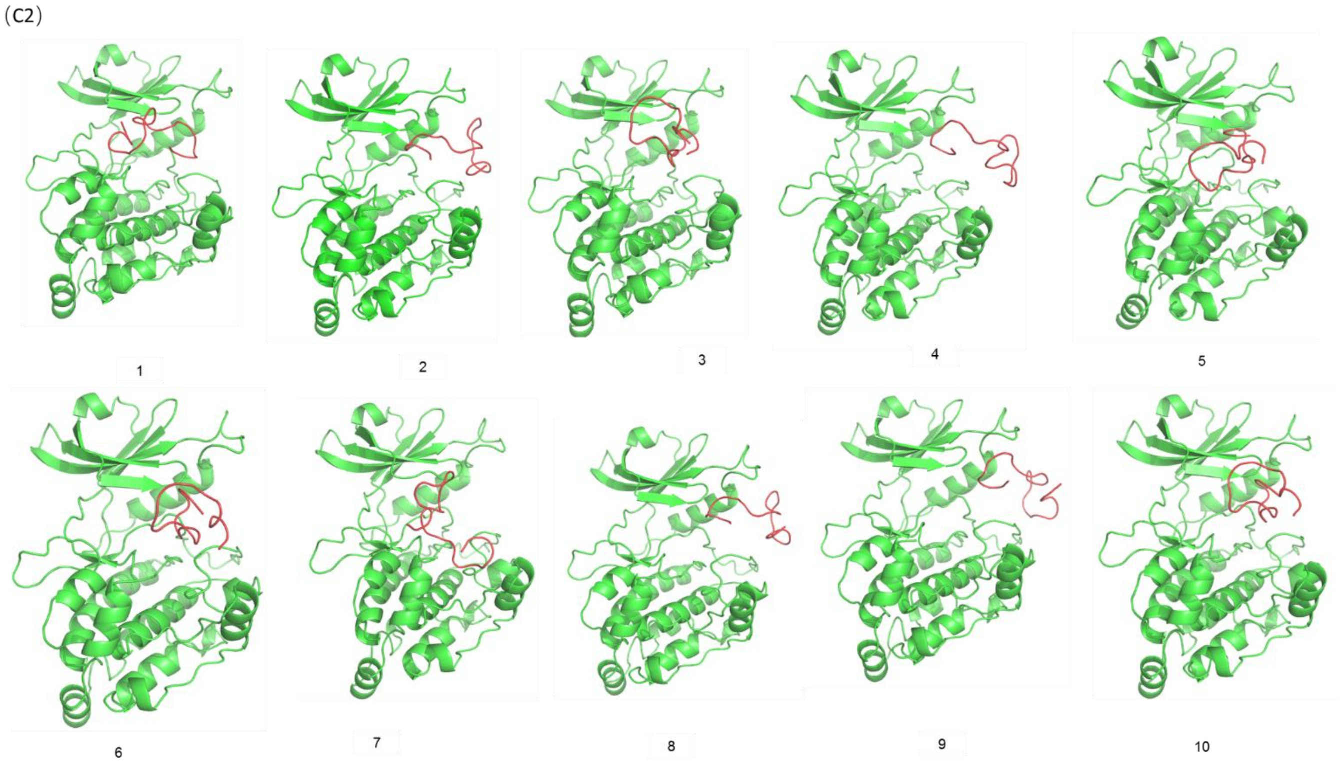

Figure S9. The docking poses of DAPK1 (PDB code 2XZS) and GluN2B-CT$_{1290-1310}$ were obtained from ZDOCK. GluN2B-CT$_{1290-1310}$ was extracted from the final 100 ns MD trajectory C.

(A3)

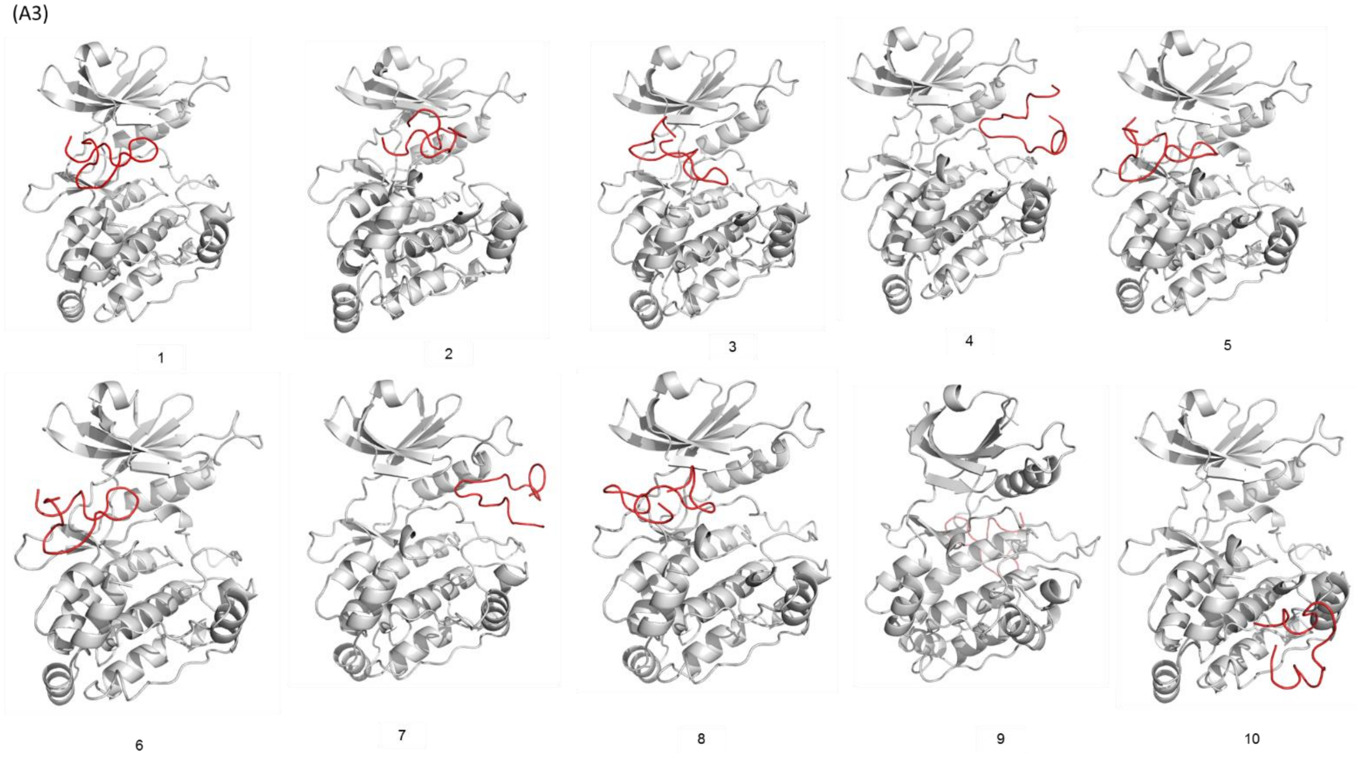

Figure S10. The docking poses of DAPK1 (PDB code 2XZS) and GluN2B-$CT_{1290-1310}$ were obtained from SwarmDock. GluN2B-$CT_{1290-1310}$ was extracted from the final 100 ns MD trajectory A.

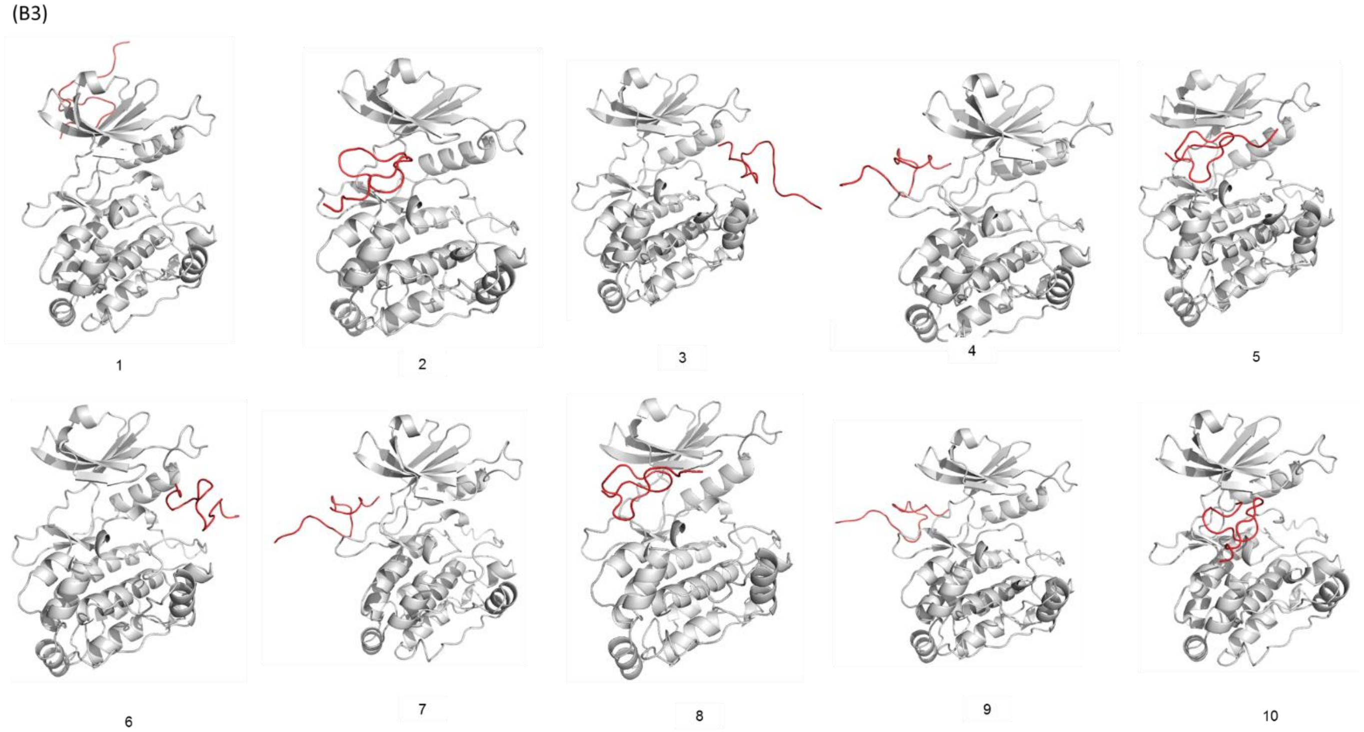

Figure S11. The docking poses of DAPK1 (PDB code 2XZS) and GluN2B-CT$_{1290\text{-}1310}$ were obtained from SwarmDock. GluN2B-CT$_{1290\text{-}1310}$ was extracted from the final 100 ns MD trajectory B.

(C3)

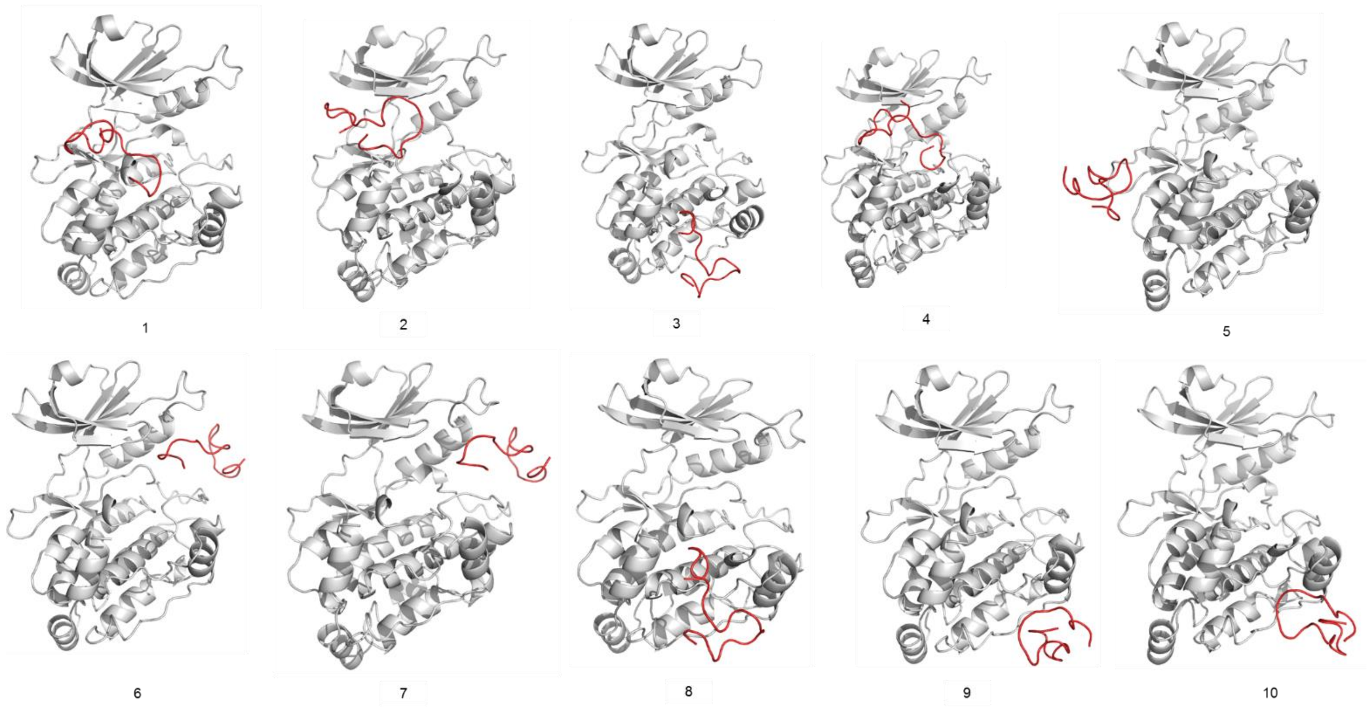

Figure S12. The docking poses of DAPK1 (PDB code 2XZS) and GluN2B-$CT_{1290\text{-}1310}$ were obtained from SwarmDock. GluN2B-$CT_{1290\text{-}1310}$ was extracted from the final 100 ns MD trajectory C.

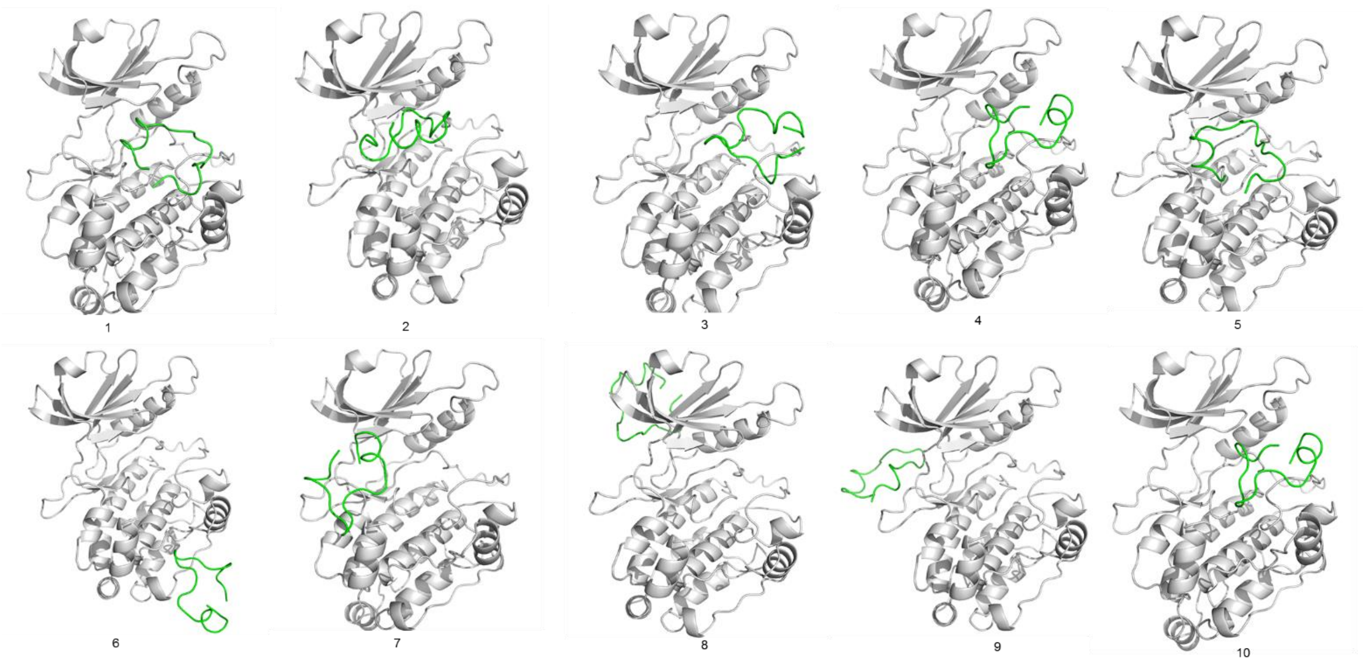

Figure S13. The docking poses of DAPK1 (PDB code 2XZS) and GluN2B-CT$_{1290\text{-}1310}$ were obtained from GRAMM-X. GluN2B-CT$_{1290\text{-}1310}$ was extracted from the first 50 ns MD trajectory A.

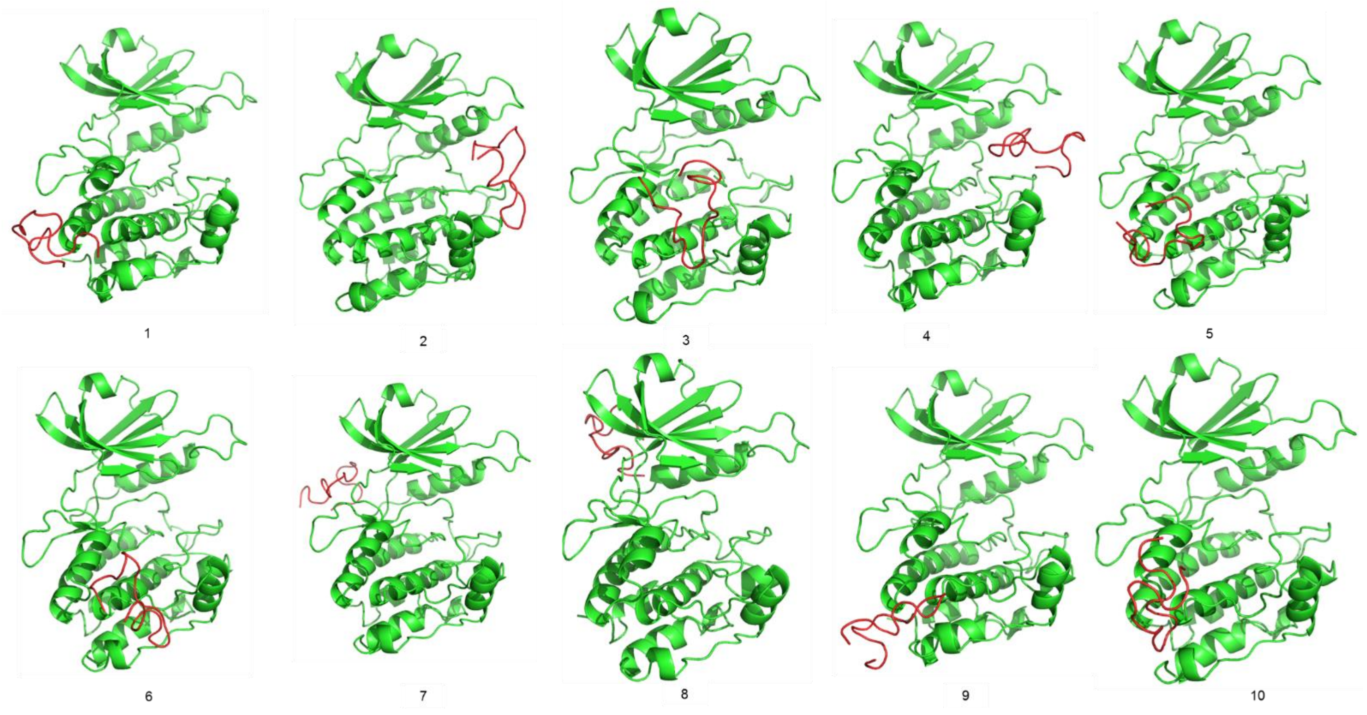

Figure S14. The docking poses of DAPK1 (PDB code 1JKS) and GluN2B-CT$_{1290\text{-}1310}$ were obtained from GRAMM-X. GluN2B-CT1290-1310 was extracted from the final 100 ns MD trajectory A.

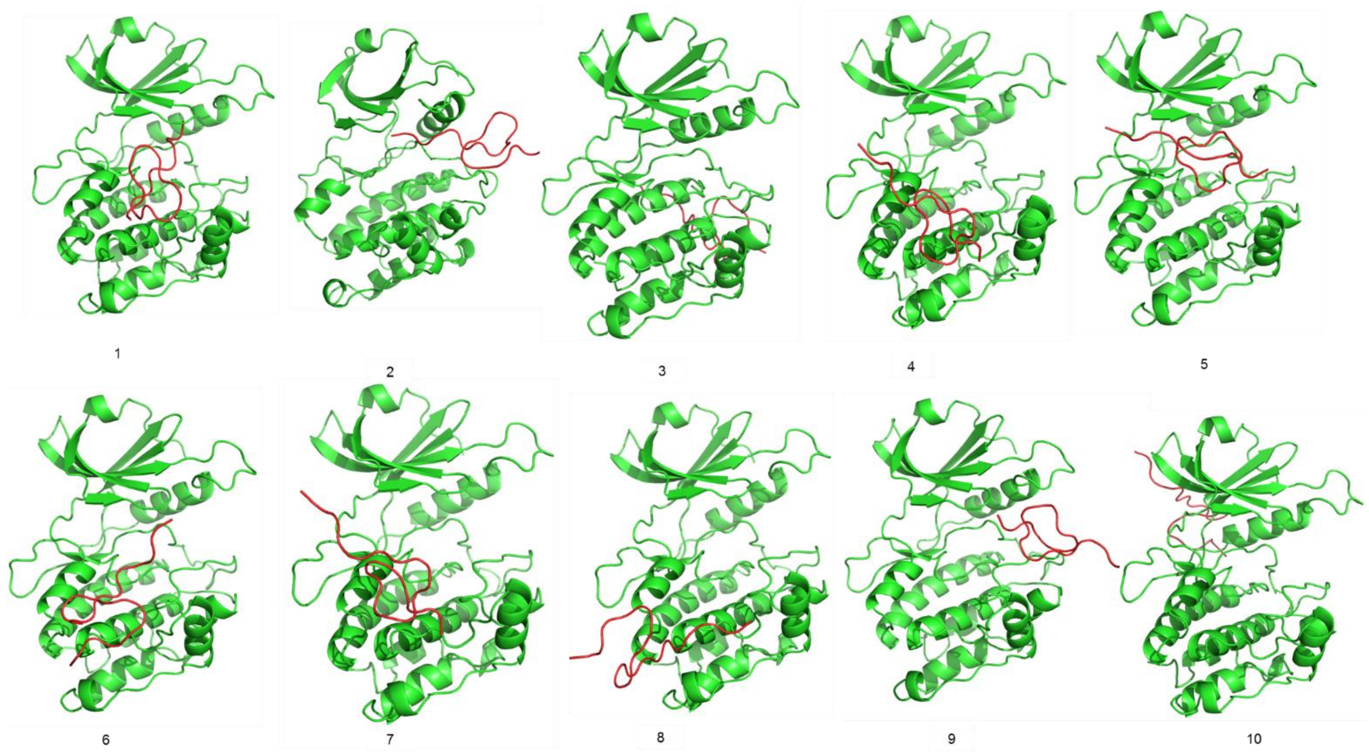

Figure S15. The docking poses of DAPK1 protein (PDB code: 1JKS) and GluN2B-$CT_{1290-1310}$ were obtained from GRAMM-X Docking sever. GluN2B-$CT_{1290-1310}$ was extracted from the final 100 ns MD trajectory B.

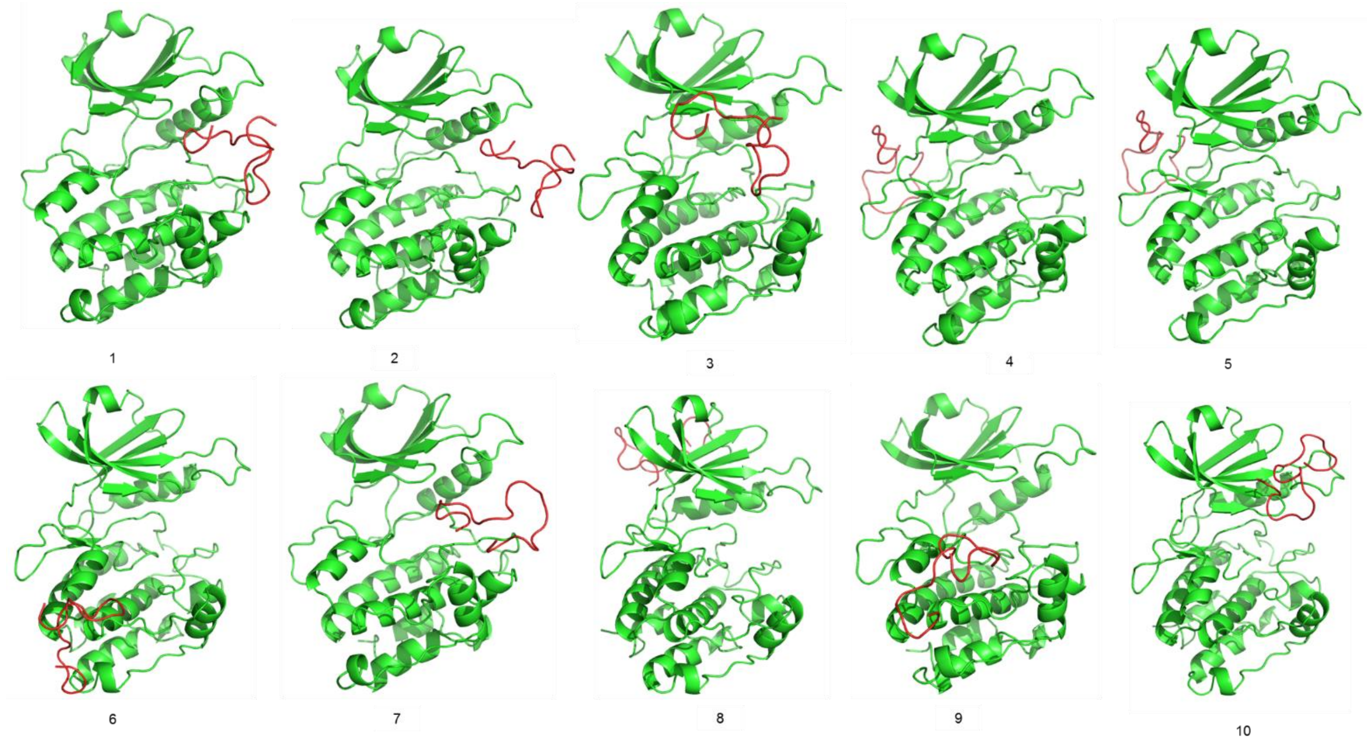

Figure S16. The docking poses of DAPK1 (PDB code 1JKS) and GluN2B-$CT_{1290-1310}$ were obtained from GRAMM-X. GluN2B-$CT_{1290-1310}$ was extracted from the final 100 ns MD trajectory C.

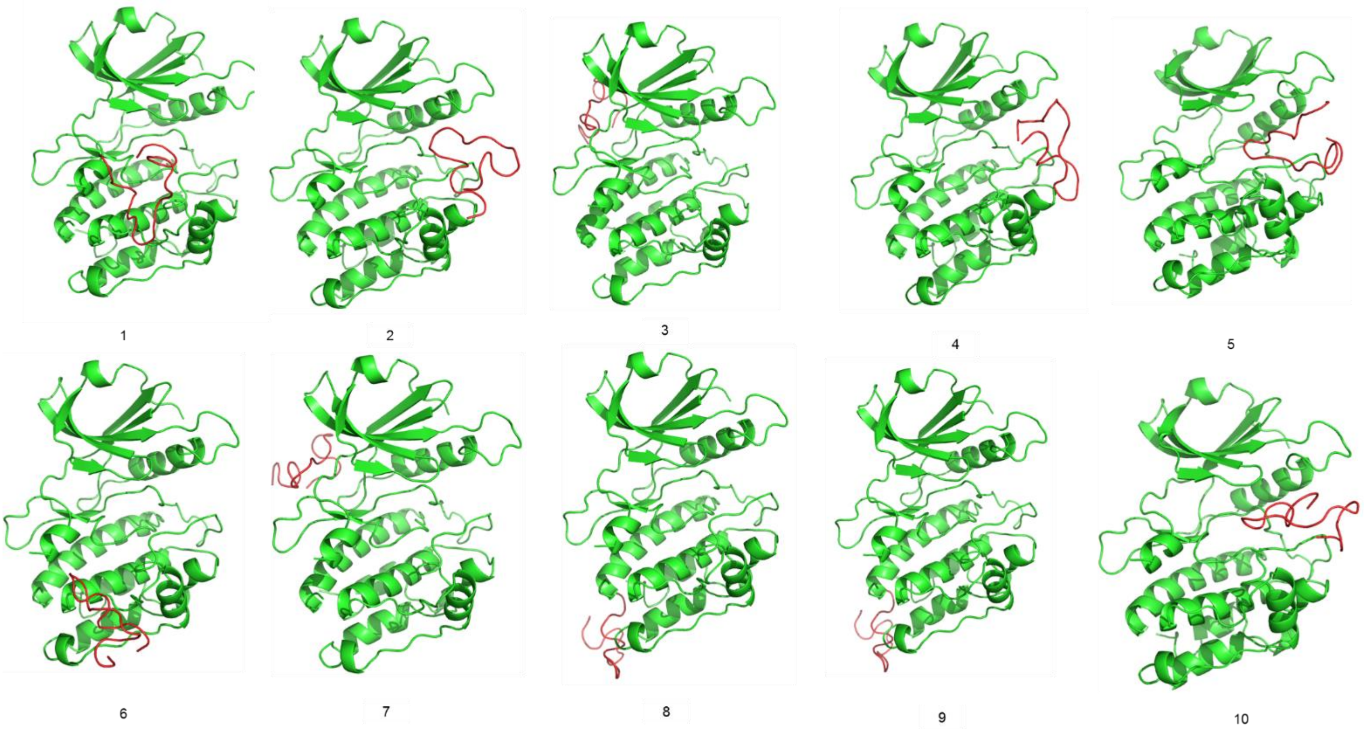

Figure S17. The docking poses of DAPK1 (PDB code 5AV4) and GluN2B-CT$_{1290-1310}$ were obtained from GRAMM-X. GluN2B-CT$_{1290-1310}$ was extracted from the final 100 ns MD trajectory A.

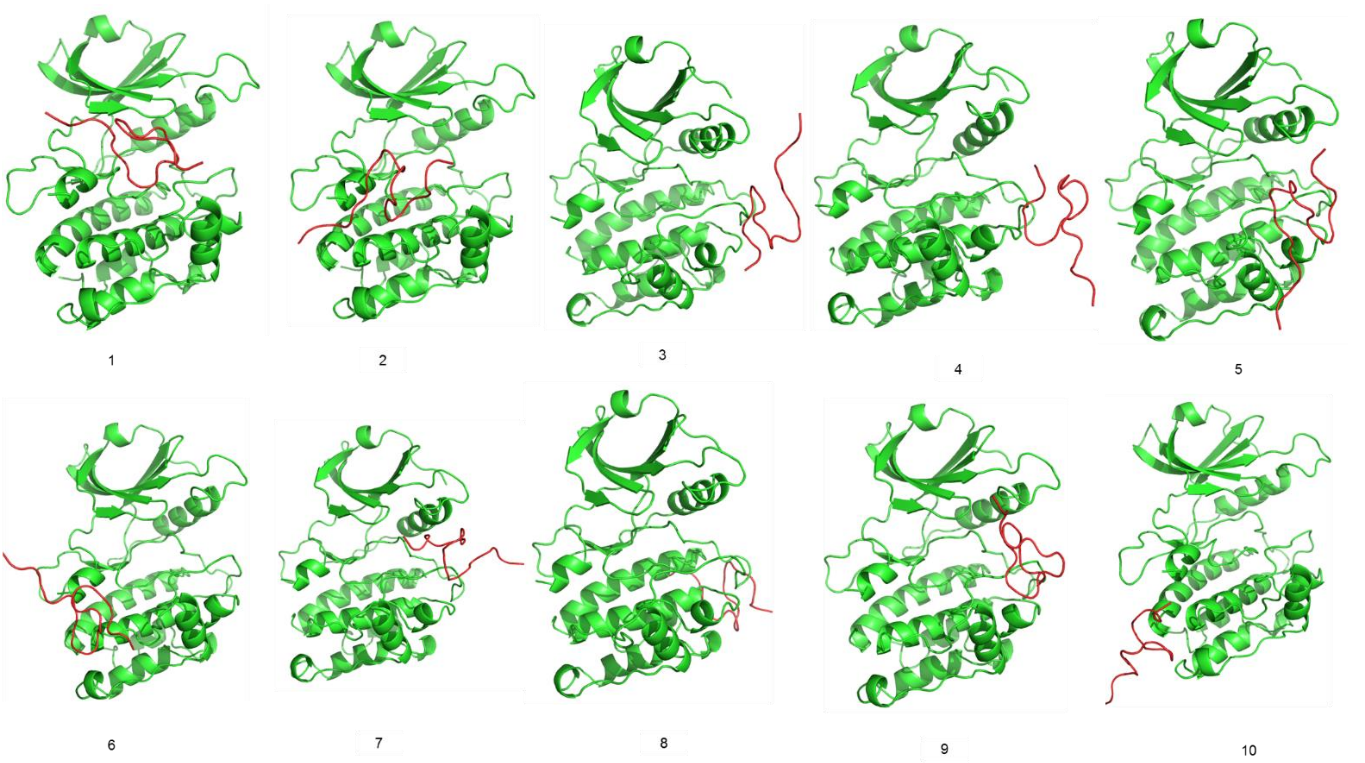

Figure S18. The docking poses of DAPK1 (PDB code 5AV4) and GluN2B-CT$_{1290-1310}$ were obtained from GRAMM-X. GluN2B-CT$_{1290-1310}$ was extracted from the final 100 ns MD trajectory B.

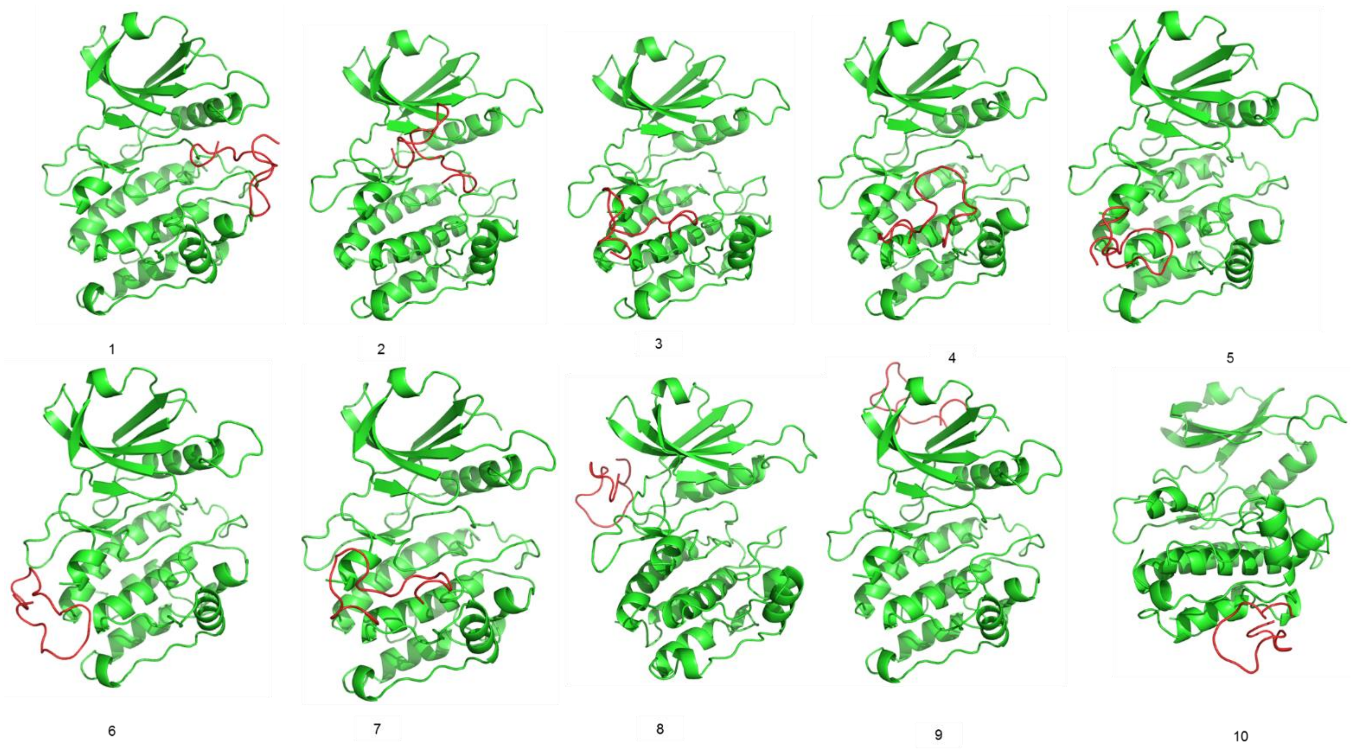

Figure S19. The docking poses of DAPK1 (PDB code 5AV4) and GluN2B-CT$_{1290\text{-}1310}$ were obtained from GRAMM-X. GluN2B-CT$_{1290\text{-}1310}$ was extracted from the final 100 ns MD trajectory C.

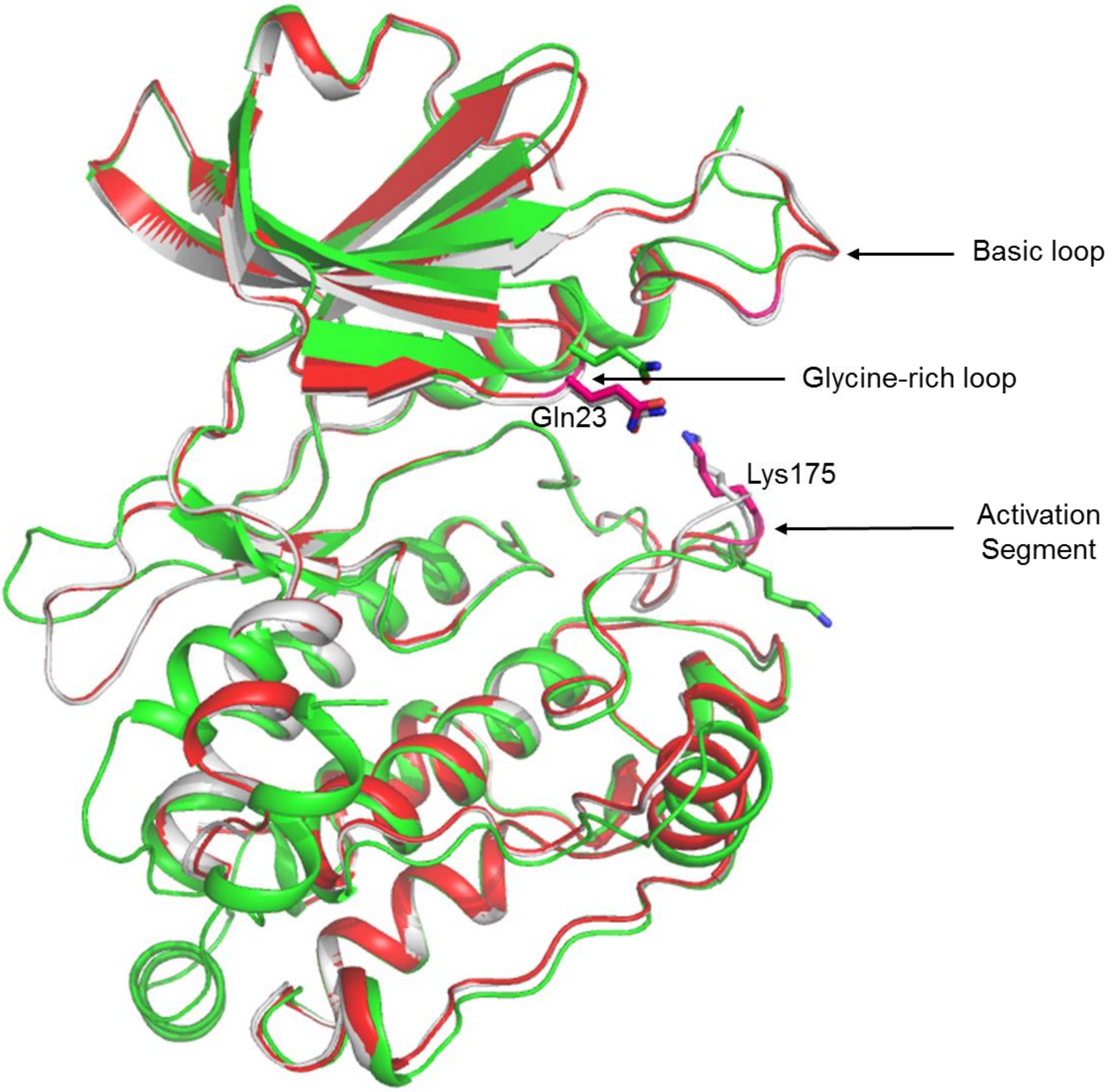

Figure S20. The superimposition of DAPK1 crystal structures of 2XZS (green ribbon),1JKS (red ribbon) and 5AV4 (gray ribbon). The residues of the 2XZS,1JKS and 5AV4 are represented by green, red and gray sticks, respectively.

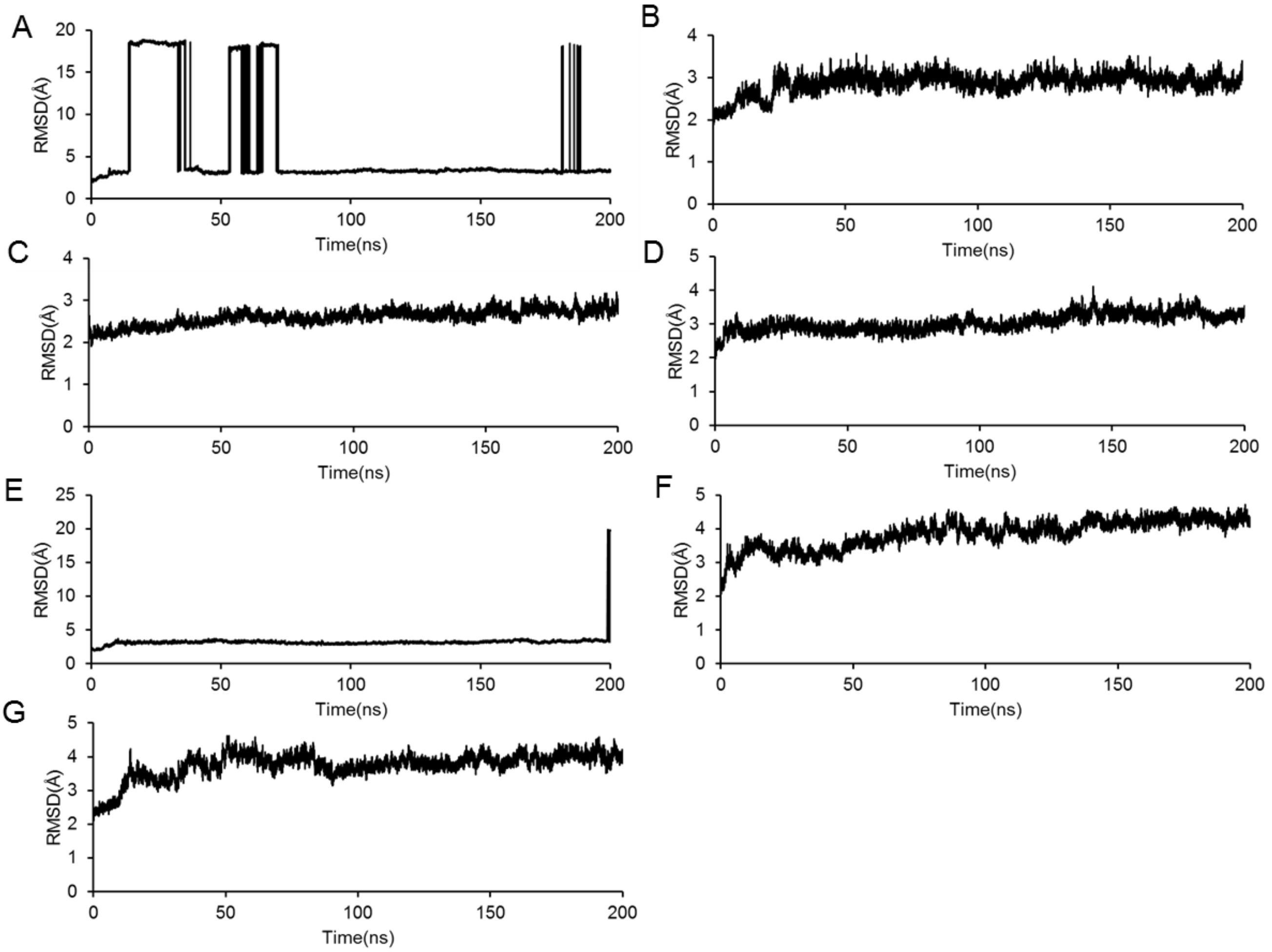

Figure S21. The monitored root mean squared deviation (RMSD) of the all atoms with respect to the initial structure during the 200 ns simulation. RMSD values of (**A**) complex 2, (**B**) complex 3, (**C**) complex 4, (**D**) complex 5, (**E**) complex 6, (**F**) complex 7 and (**G**) complex 8.

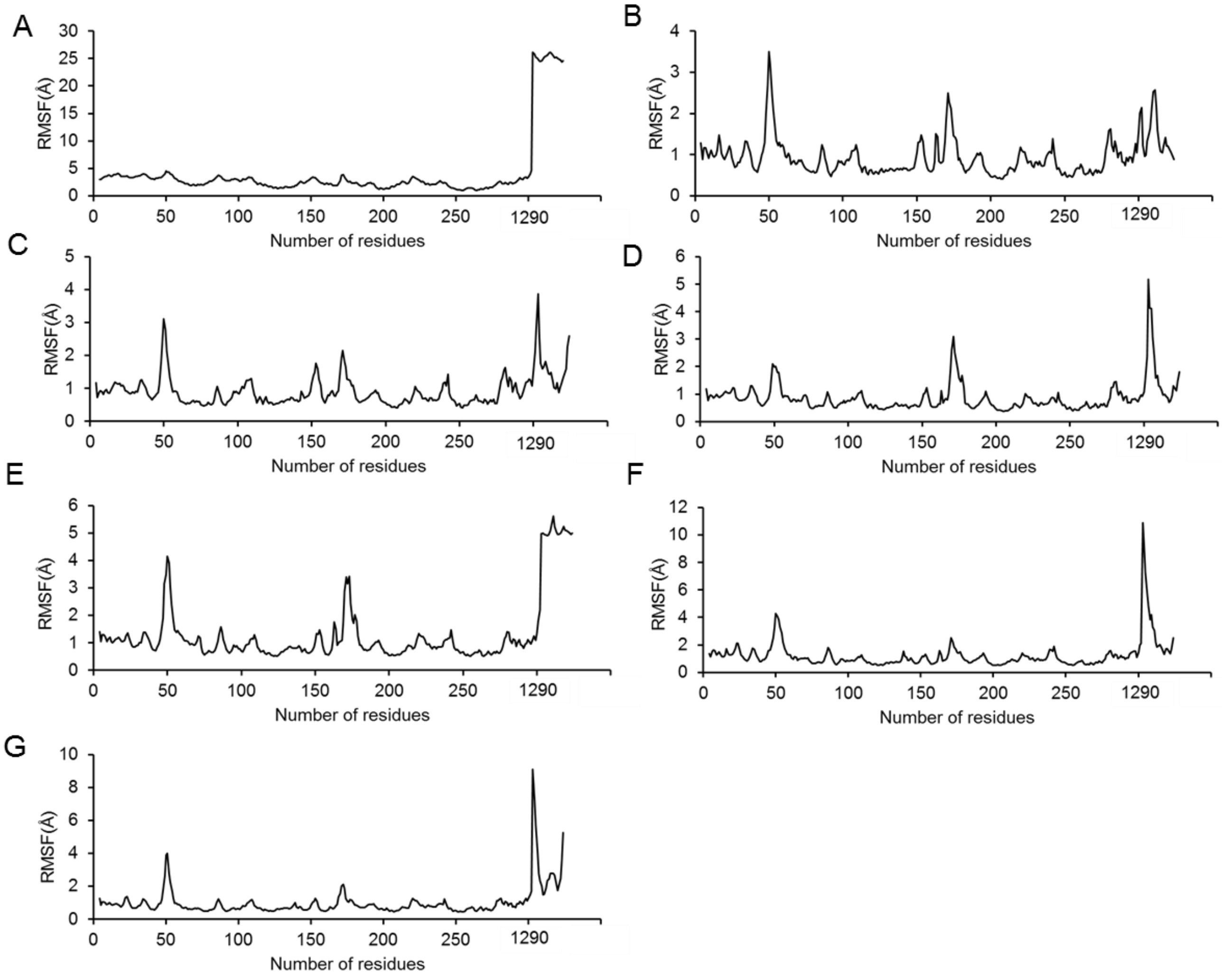

Figure S22. The RMSF value of backbone atoms of complexes during the 200 ns MD simulation. RMSD values of (**A**) complex 2, (**B**) complex 3, (**C**) complex 4, (**D**) complex 5, (**E**) complex 6, (**F**) complex 7 and (**G**) complex 8.

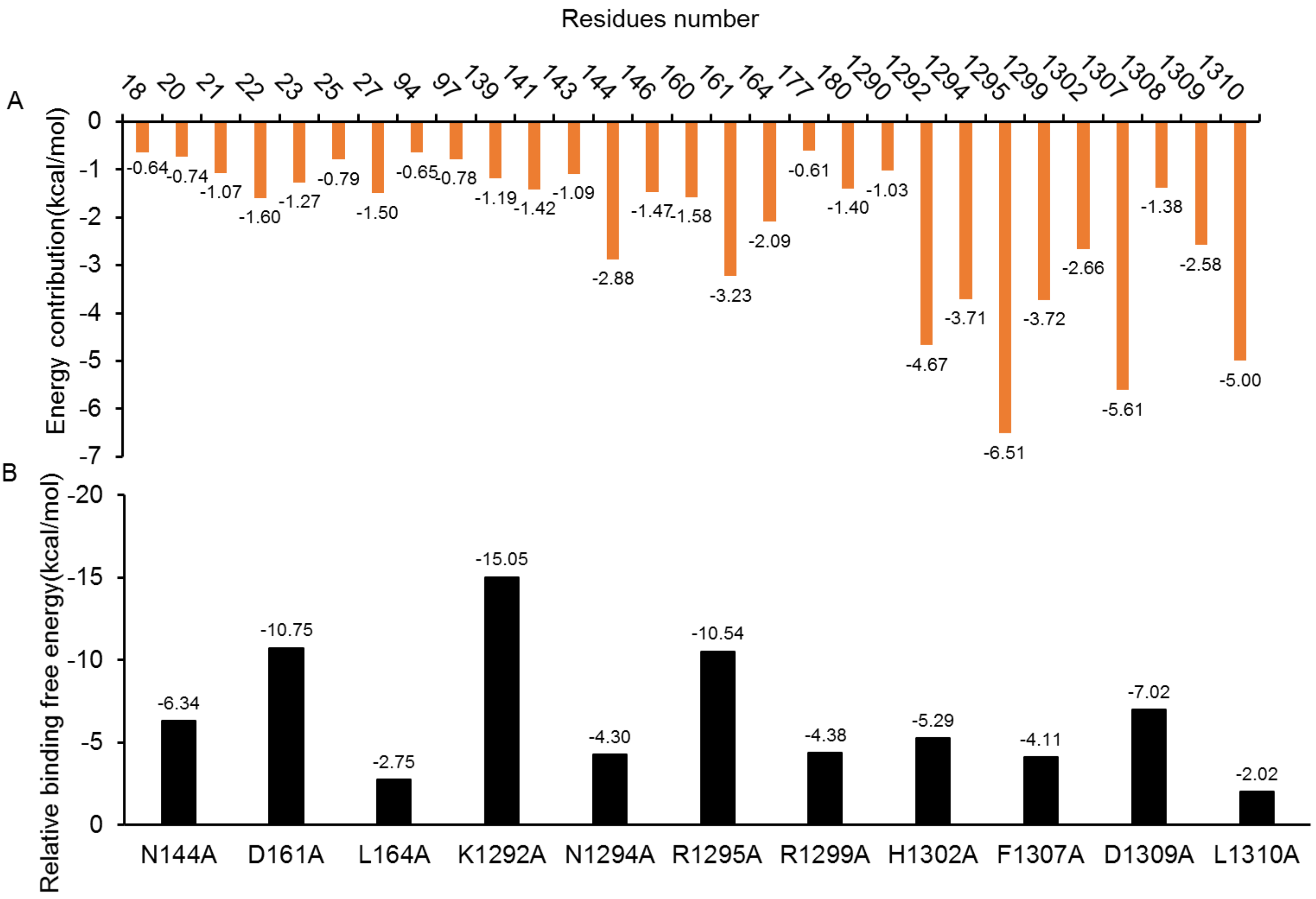

Figure S23. (**A**) Per-residue binding energy decomposition of the predicted GluN2B-CT$_{1290–1310}$/DAPK1 complex 3. The energy contribution (the absolute value) larger than 0.60 kcal/mol to at least one studied to the binding of GluN2B-CT$_{1290–1310}$/DAPK1 was displayed. The orange bar shows the residues with the absolute binding free energy value more than 0.60 kcal/mol. (**B**) Alanine scanning analyses of the predicted GluN2B-CT$_{1290–1310}$/DAPK1 complex 3.

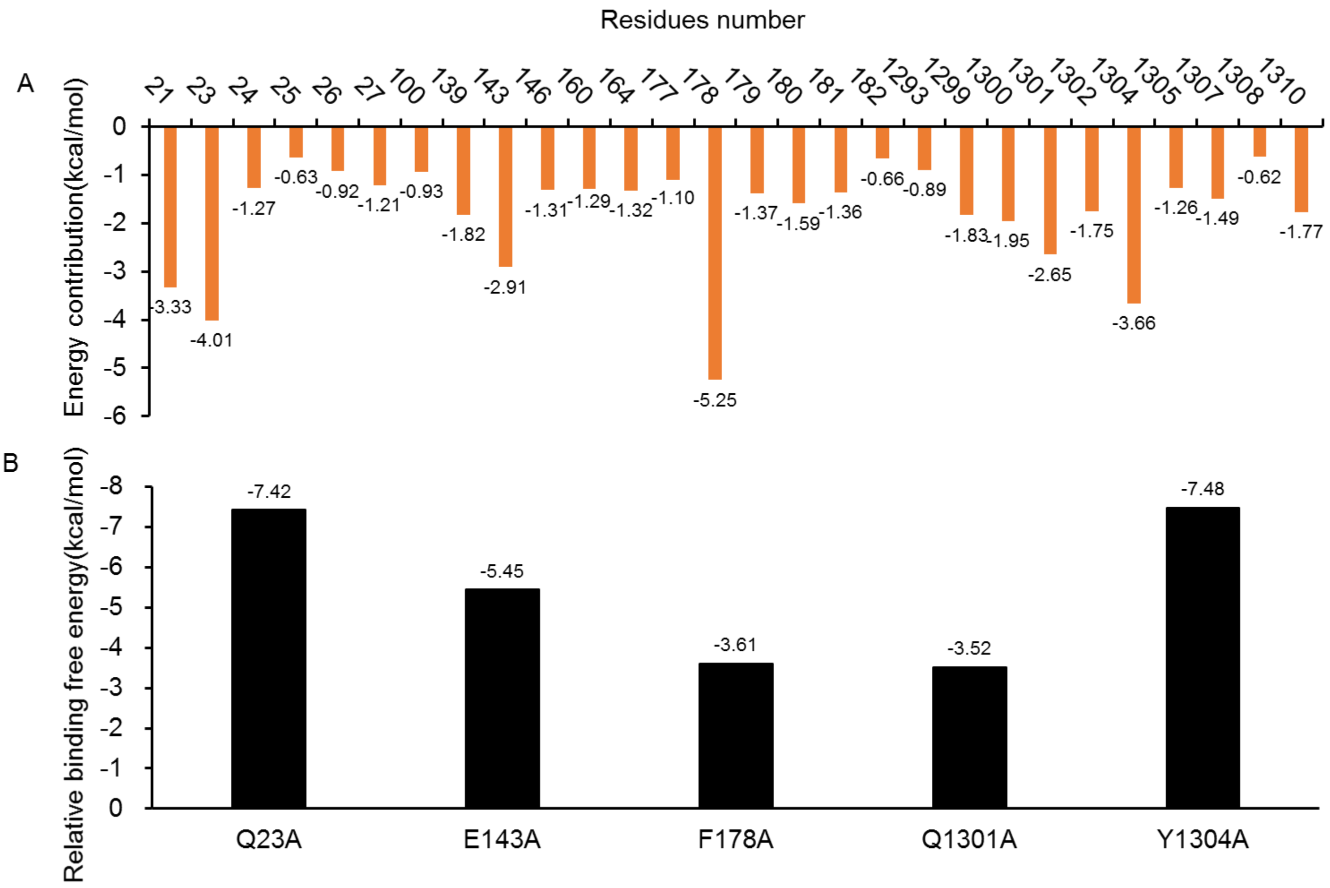

Figure S24. (**A**) Per-residue binding energy decomposition of the predicted GluN2B-CT$_{1290–1310}$/DAPK1 complex 4. The energy contribution (the absolute value) larger than 0.60 kcal/mol to at least one studied to the binding of GluN2B-CT$_{1290–1310}$/DAPK1 was displayed. The orange bar shows the residues with the absolute binding free energy value more than 0.60 kcal/mol. (**B**) Alanine scanning analyses of the predicted GluN2B-CT$_{1290–1310}$/DAPK1 complex 4.

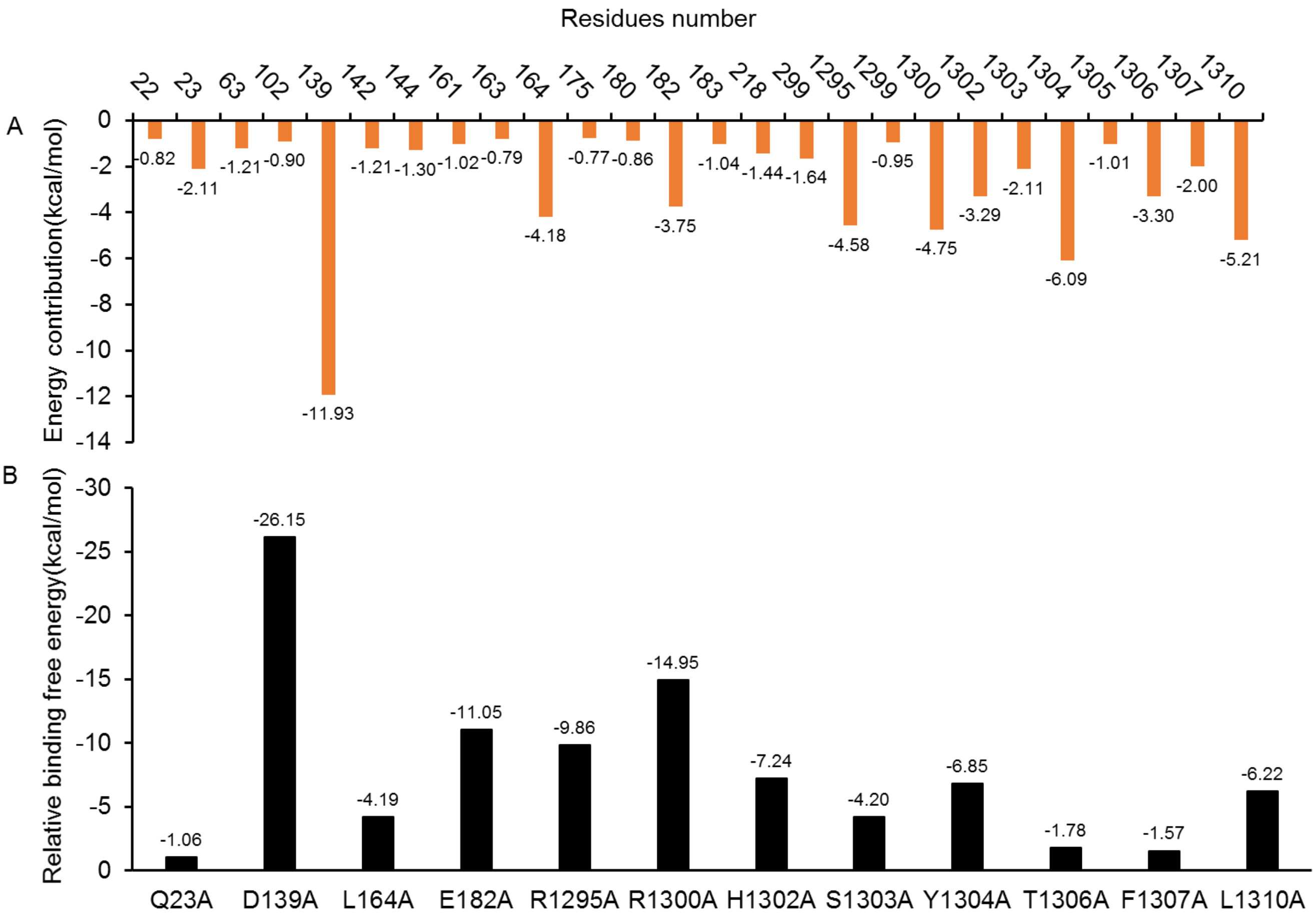

Figure S25. (**A**) Per-residue binding energy decomposition of the predicted GluN2B-CT$_{1290–1310}$/DAPK1 complex 5. The energy contribution (the absolute value) larger than 0.60 kcal/mol to at least one studied to the binding of GluN2B-CT$_{1290–1310}$/DAPK1 was displayed. The orange bar shows the residues with the absolute binding free energy value more than 0.60 kcal/mol. (**B**) Alanine scanning analyses of the predicted GluN2B-CT$_{1290–1310}$/DAPK1 complex 5.

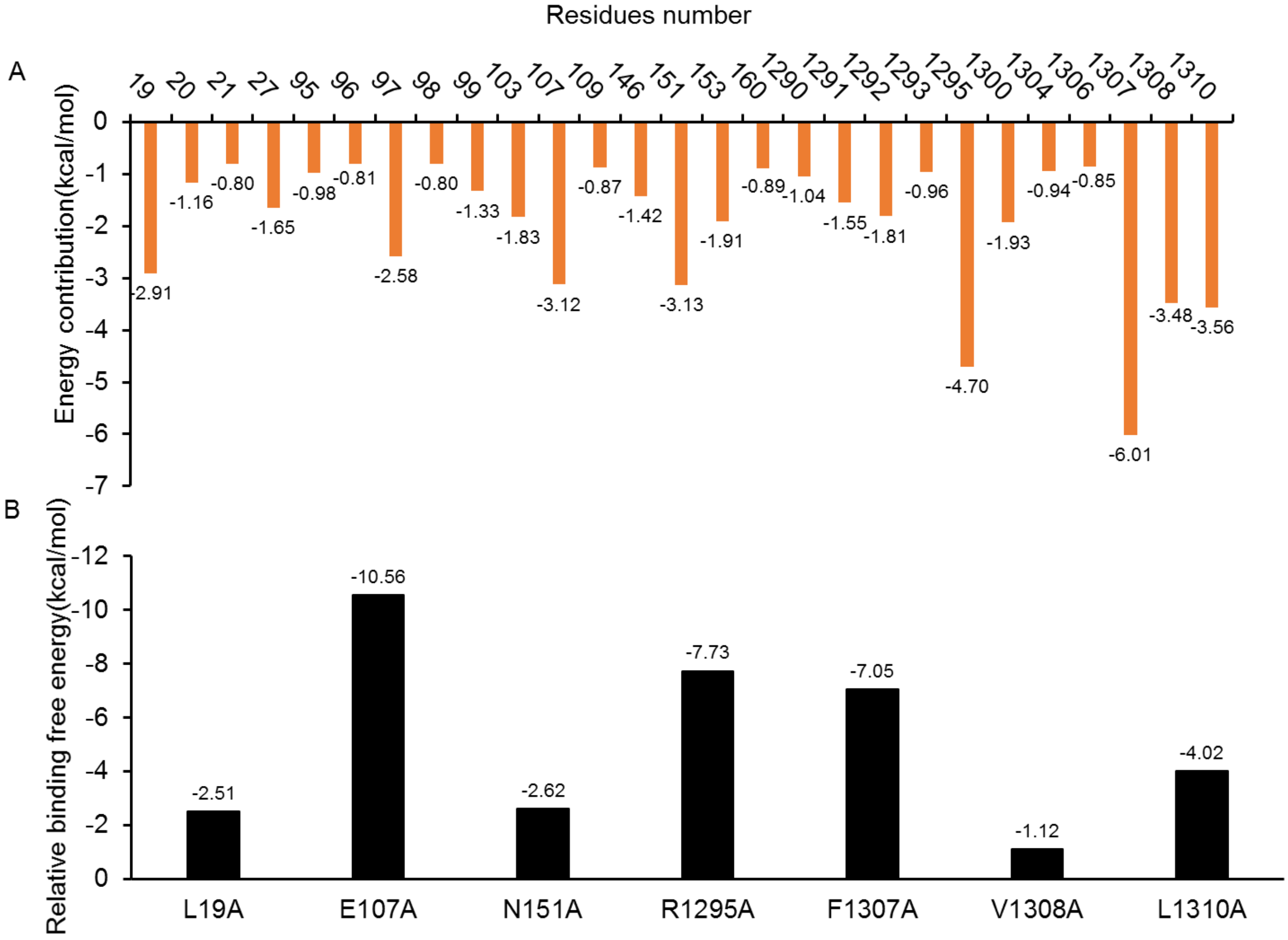

Figure S26. (**A**) Per-residue binding energy decomposition of the predicted GluN2B-CT$_{1290–1310}$/DAPK1 complex 7. The energy contribution (the absolute value) larger than 0.60 kcal/mol to at least one studied to the binding of GluN2B-CT$_{1290–1310}$/DAPK1 was displayed. The orange bar shows the residues with the absolute binding free energy value more than 0.60 kcal/mol. (**B**) Alanine scanning analyses of the predicted GluN2B-CT$_{1290–1310}$/DAPK1 complex 7.

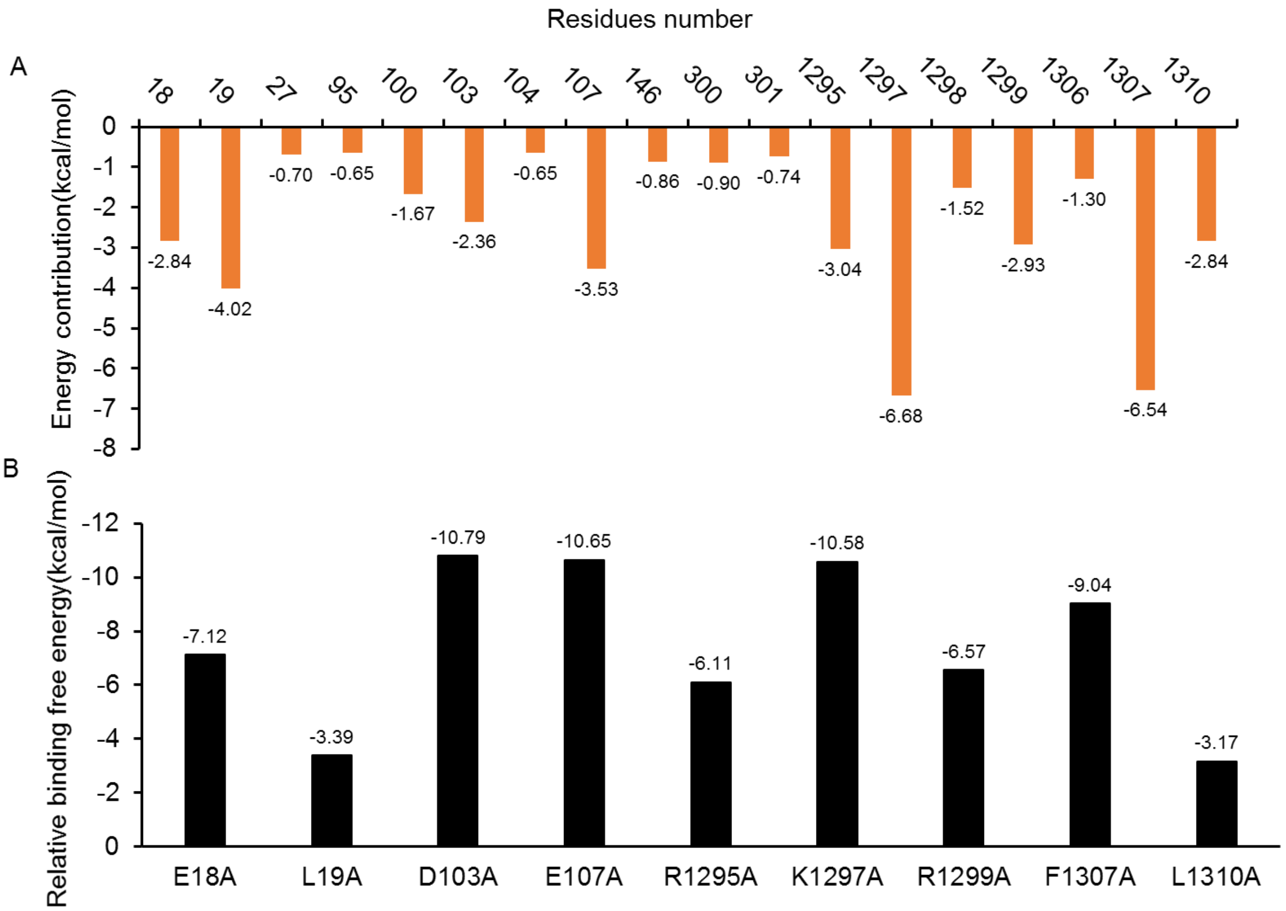

Figure S27. (**A**) Per-residue binding energy decomposition of the predicted GluN2B-CT$_{1290–1310}$/DAPK1 complex 8. The energy contribution (the absolute value) larger than 0.60 kcal/mol to at least one studied to the binding of GluN2B-CT$_{1290–1310}$/DAPK1 was displayed. The orange bar shows the residues with the absolute binding free energy value more than 0.60 kcal/mol. (**B**) Alanine scanning analyses of the predicted GluN2B-CT$_{1290–1310}$/DAPK1 complex 8.

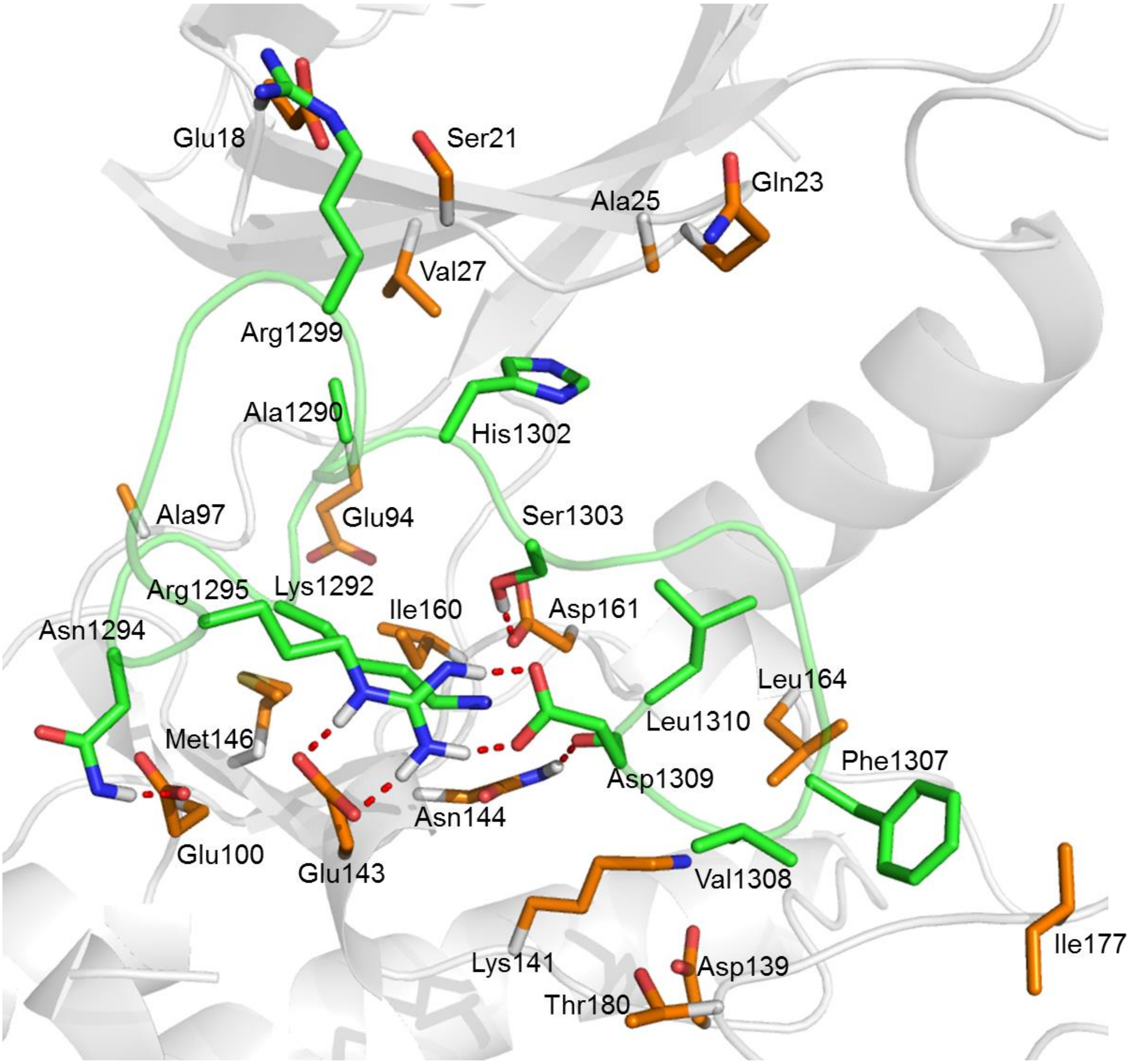

Figure S28. The predicted key interactions between the DAPK1 and the GluN2B-CT$_{1290–1310}$ peptide in the predicted complex 3. The DAPK1 is shown in a grey cartoon while the GluN2B-CT$_{1290–1310}$ is shown as green cartoon. The C-terminal residues of the DAPK1 and the GluN2B-CT$_{1290–1310}$ binding site residues are represented by orange and green sticks, respectively. Red dashed lines represent the hydrogen bond.

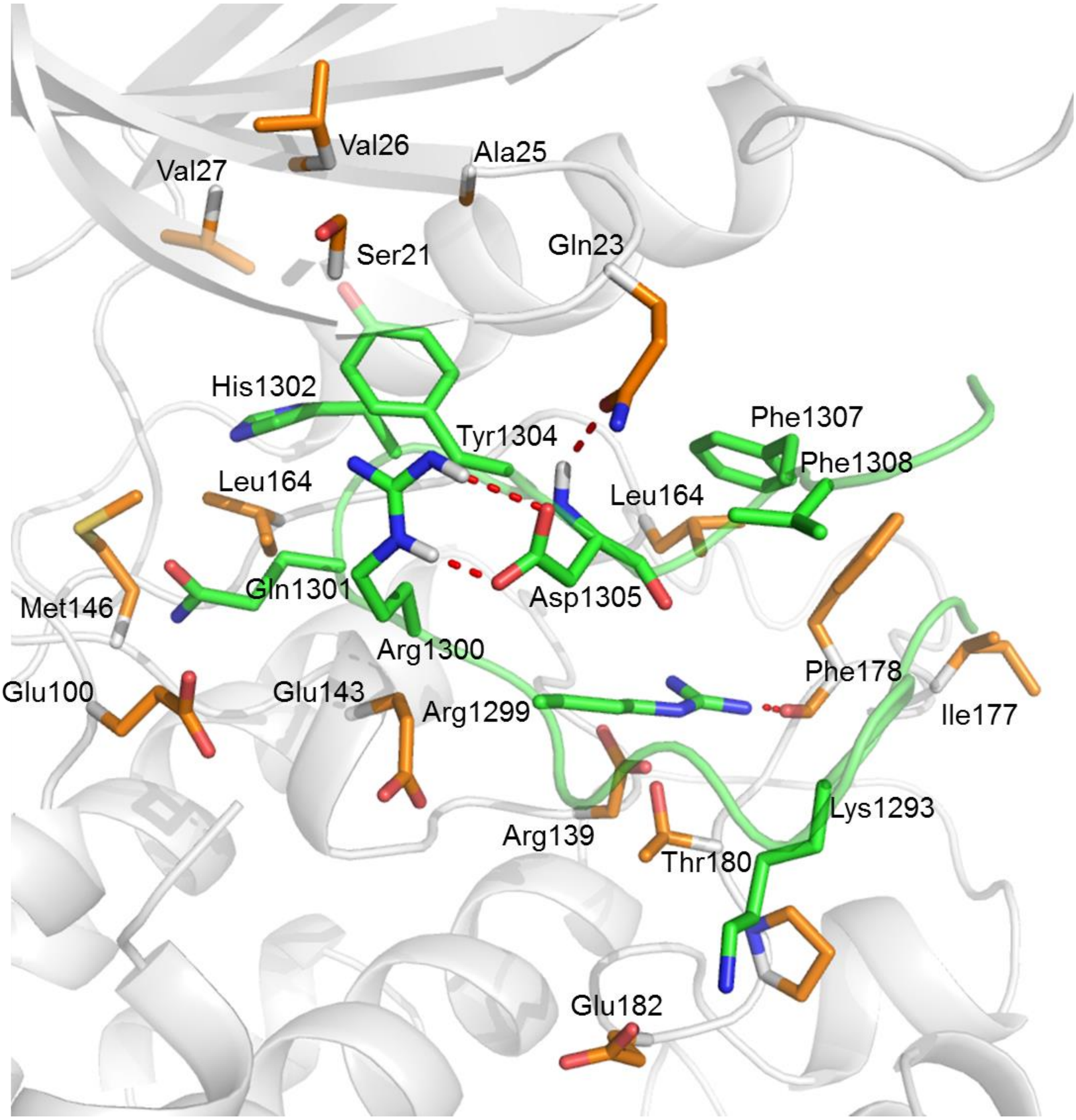

Figure S29. The predicted key interactions between the DAPK1 and the GluN2B-CT$_{1290–1310}$ peptide in the predicted complex 4. The DAPK1 is shown in a grey cartoon while the GluN2B-CT$_{1290–1310}$ is shown as green cartoon. The C-terminal residues of the DAPK1 and the GluN2B-CT$_{1290–1310}$ binding site residues are represented by orange and green sticks, respectively. Red dashed lines represent the hydrogen bond.

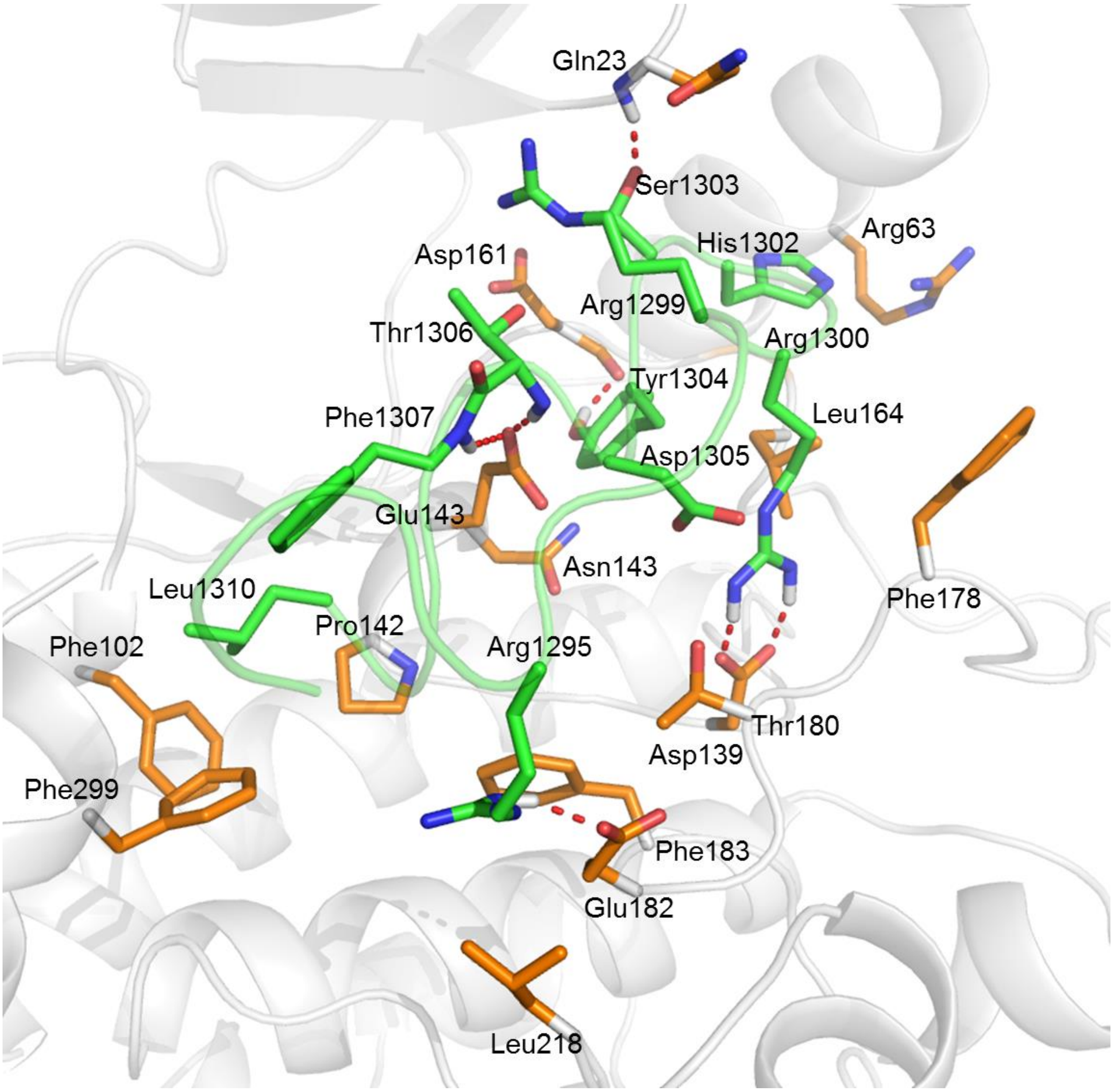

Figure S30. The predicted key interactions between the DAPK1 and the GluN2B-CT$_{1290–1310}$ peptide in the predicted complex 5. The DAPK1 is shown in a grey cartoon while the GluN2B-CT$_{1290–1310}$ is shown as green cartoon. The C-terminal residues of the DAPK1 and the GluN2B-CT$_{1290–1310}$ binding site residues are represented by orange and green sticks, respectively. Red dashed lines represent the hydrogen bond.

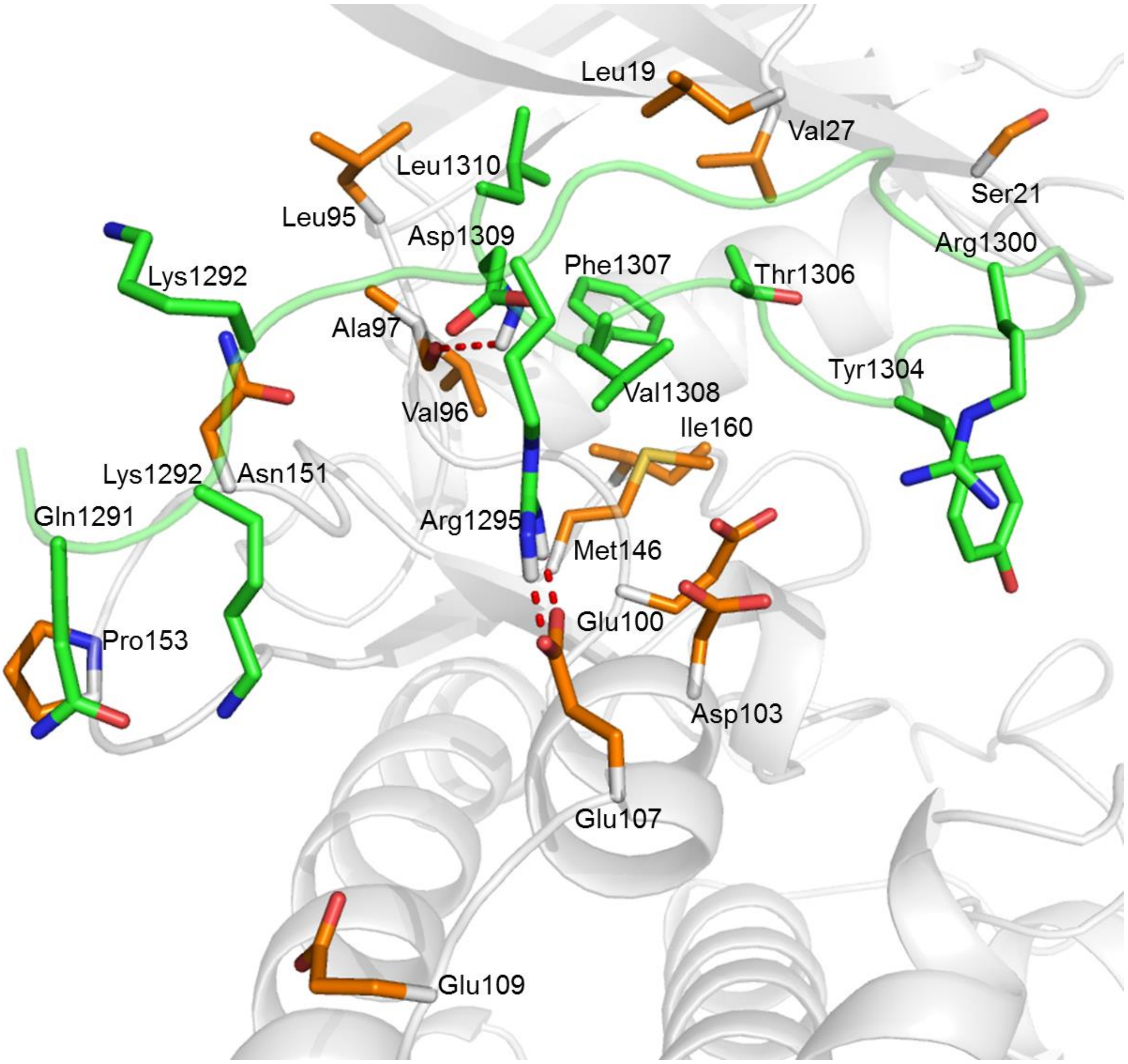

Figure S31. The predicted key interactions between the DAPK1 and the GluN2B-CT$_{1290–1310}$ peptide in the predicted complex 7. The DAPK1 is shown in a grey cartoon while the GluN2B-CT$_{1290–1310}$ is shown as green cartoon. The C-terminal residues of the DAPK1 and the GluN2B-CT$_{1290–1310}$ binding site residues are represented by orange and green sticks, respectively. Red dashed lines represent the hydrogen bond.

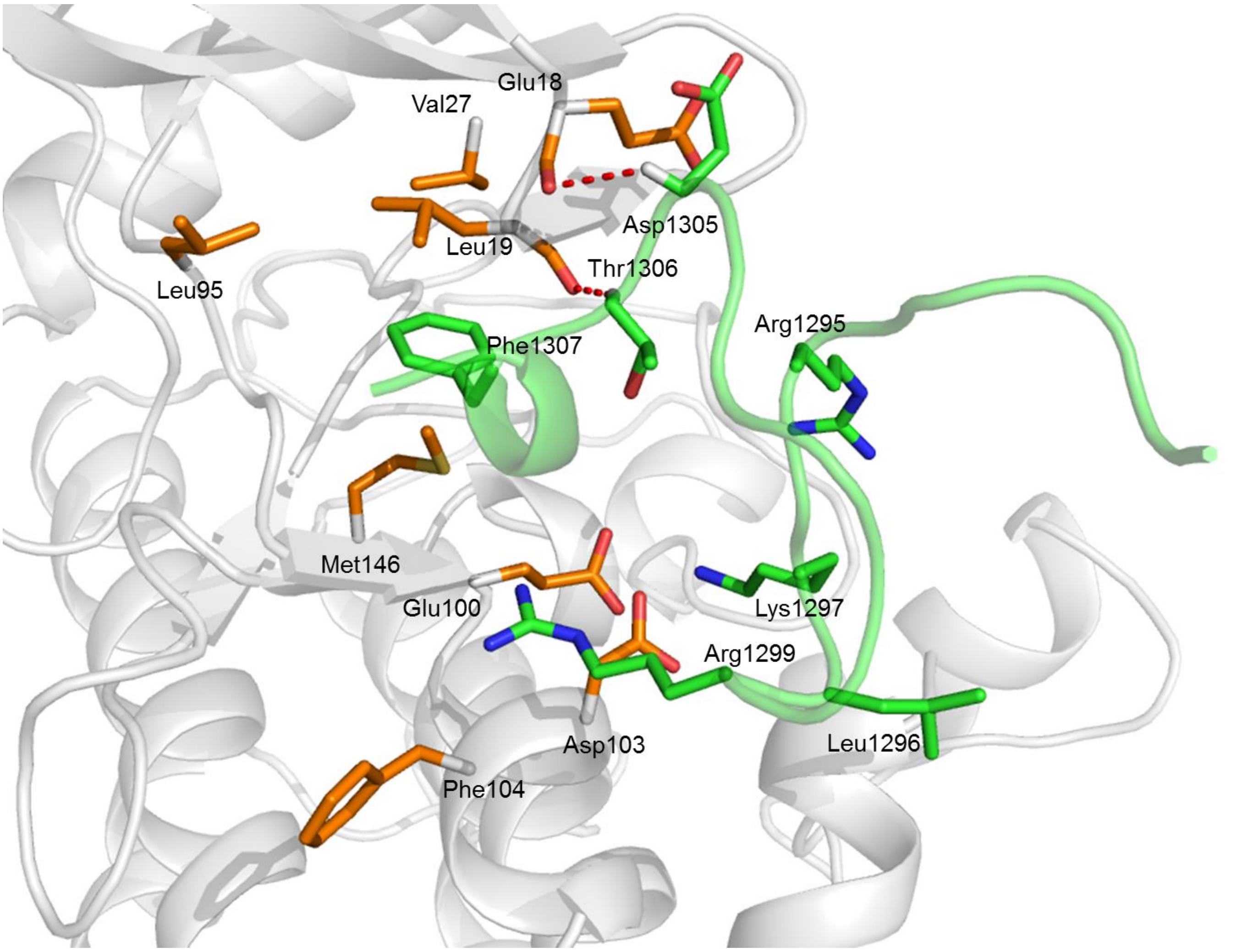

Figure S32. The predicted key interactions between the DAPK1 protein and the GluN2B-CT$_{1290–1310}$ peptide in the predicted complex 8. The DAPK1 is shown in a grey cartoon while the GluN2B-CT$_{1290–1310}$ is shown as green cartoon. The C-terminal residues of the DAPK1 and the GluN2B-CT$_{1290–1310}$ binding site residues are represented by orange and green sticks, respectively. Red dashed lines represent the hydrogen bond.
